# The Mental Health Impact of the COVID‐19 Pandemic on Health and Social Care Workers

**DOI:** 10.1002/hec.70090

**Published:** 2026-03-09

**Authors:** Victoria Serra‐Sastre, Jaime Pinilla, Wasana Kalansooriya

**Affiliations:** ^1^ City St George's University of London London UK; ^2^ London School of Economics and Political Science London UK; ^3^ Office of Health Economics London UK; ^4^ Universidad de Las Palmas de Gran Canaria Las Palmas de Gran Canaria Spain; ^5^ King's College London London UK

**Keywords:** covid‐19, difference‐in‐differences estimator, health and social care worker, keyworker, mental health

## Abstract

The COVID‐19 pandemic placed exceptional strain on essential services, raising urgent concerns about the mental well‐being of workers in critical sectors. This study examines the short‐ and medium‐term effects of the COVID‐19 pandemic on the mental health of health and social care (HSC) workers in the UK relative to other occupational groups. Using data from the UK Household Longitudinal Study and measuring mental health via the General Health Questionnaire (GHQ), we apply a difference‐in‐differences strategy, where both groups could be treated only in the second period (a pre‐post design), to investigate whether HSC workers experienced distinct mental health trajectories compared to other key workers (KWs) and workers in non‐essential sectors (non‐KWs). The results for the immediate post‐pandemic period (April–November 2020) show no significant differences in mental health for HSC workers compared with either comparator worker groups. Medium‐term outcomes remained statistically insignificant across occupational comparisons. Additional analyses of individual GHQ items and potential mechanisms (financial stability and social isolation) suggest limited heterogeneous effects for each worker group using yearly data. While all studied groups exhibited some deterioration in mental health after 2020, HSC workers' trajectories largely mirrored those of other KWs and non‐KWs, suggesting that factors such as stable employment and financial security may have cushioned the psychological impact for this sector.

## 1 Introduction

In this paper, we examine the impact of the COVID‐19 pandemic on the mental health of health and social care (HSC) workers, comparing their mental health outcomes relative to other occupational groups in the economy. HSC workers, as a workforce category, belong to the wider classification of keyworkers (KWs). KWs provide essential services crucial for the functioning of society, especially in times of crisis such as the COVID‐19 pandemic. In addition to HSC workers, the UK government's definition of KWs includes those employed in education and childcare, utilities and communication, food production and distribution and other essential goods, transport, key public services, public safety and national security, and national and local government (Office for National Statistics 2020). For the purpose of this paper, mental health trajectories of HSC workers are examined within the broader context of deteriorating mental health observed across the wider population (Banks and Xu 2020; Pierce et al. 2020; Kromydas et al. 2022).

Prior research has compared KWs with non‐KWs, documenting worse mental health among essential workers as a whole (Bu et al. 2022; Ayling et al. 2020; May et al. 2021). However, the KW population is heterogeneous, including sectors such as HSC, education and transport, among many others. Aggregating across these groups may obscure meaningful variation in how the pandemic affected mental health across groups. HSC workers bore a particularly heavy burden. Increased workloads, high‐pressure working conditions, and the emotional toll of witnessing high patient mortality were among the main factors affecting the mental well‐being of the HSC workforce (NHS Confederation 2020). Mental health conditions, including anxiety, stress and depression accounted for 27.4% of all illness‐related absences among NHS staff, translating into nearly 510,800 full‐time equivalent days lost (NHS Digital 2023). This is in contrast to the wider economy, where approximately 8% of absences from work were due to mental health factors (Office for National Statistics 2022).

Studies examining the impact of COVID‐19 on the mental health of HSC workers largely focus on indicators of mental health during and after the pandemic, failing to capture the existing dynamic nature of mental health prior to the pandemic (Bu et al. 2022; Toh et al. 2021; Mercado et al. 2022; Doleman et al. 2023; Guan et al. 2020; van der Plaat et al. 2021; Lang et al. 2020; Kotera et al. 2022; Søvold et al. 2021). Mental health distress can evolve, with initial resilience potentially giving way to longer‐term distress or vice versa. Using a longitudinal panel data approach, our study design addresses these critical gaps and provides a more nuanced understanding by tracking changes in mental health over time before and after the onset of the pandemic. Our longitudinal design helps to adjust for time‐invariant unobserved confounding, although residual confounding from time‐varying factors might persist (Goodman‐Bacon and Marcus 2020). Consequently, our approach provides a more credible counterfactual for workers' mental health trajectories, allowing us to disentangle short‐ and medium‐term differential changes of the pandemic with greater causal validity than existing studies.

In this paper, our interest lies primarily in the mental health of HSC workers. However, this occupational group is nested within the broader classification of KWs. Based on this classification, we first examine differential effects between HSC KWs and other KWs. We next compare the mental health of HSC KWs with that of non‐essential workers (non‐KWs). For the empirical analysis we employ the UK Household Longitudinal Study (UKHLS), a panel dataset tracking the lives of individuals and their households. We use a difference‐in‐ differences (DID) strategy, where our main outcome variable of interest is worker's mental health measured using several definitions based on the General Health Questionnaire (GHQ).

Overall, we find no differential effects for HSC KWs. Amidst a general decrease in population mental health, our findings suggest the mental health of HSC workers is in decline in line with that of other worker groups. A possible explanation is that financial stability and social contact outweighed the negative effects that working in the HSC sector had during the pandemic. Our results are robust to several specifications, including the use of data by waves or years, restricting the sample to those that were continuously employed and had no prior history of mental distress before the pandemic, several classifications of worker groups and using alternative measures of mental health.

Although the acute phase of the pandemic has passed, studying its impact on mental health remains directly relevant. COVID‐19 has left a durable imprint on population mental health through both indirect societal pathways and direct clinical channels, underscoring the importance of continued analysis of trajectories and sub‐group heterogeneity.^1^ The mechanisms through which the pandemic affected mental health—prolonged uncertainty, disruption of services and routines, occupational exposure and strain, and differential vulnerability by age, gender, health status, and socioeconomic position—are not unique to pandemics (Boden et al. 2021; Penninx et al. 2022). They overlap with pathways observed in other large‐scale shocks (e.g., economic downturns or natural disasters) and map onto a framework that links social determinants and crisis stressors to mental health outcomes and scalable interventions (Penninx et al. 2022). As such, the evidence assembled during the COVID‐19 pandemic offers general lessons for preparedness and response to future system‐wide shocks.

The remainder of this paper is organized as follows: Section 2 reviews the literature of the effects of COVID‐19 on mental health across different worker categories. We describe the data used for analysis in Section 3 and the identification strategy in Section 4. The main results, extensions and robustness checks are presented in Section 5. Section 6 concludes and discusses the implications of our results.

## 2 Literature

The COVID‐19 pandemic has substantially deteriorated mental health outcomes by exacerbating pre‐existing conditions and diminishing psychological well‐being among previously healthy populations, with evidence indicating that these effects have persisted beyond the acute phase of the pandemic (García‐Prado et al. 2022; Swaziek and Wozniak 2020; Murphy et al. 2021; Cowden et al. 2021; Quintana‐Domeque and Proto 2022). Principal contributors to this decline include fear of viral infection (Usher et al. 2020; Sloan et al. 2021; Alimoradi et al. 2022), economic and food insecurity (Kämpfen et al. 2020; Swaziek and Wozniak 2020; Nagata et al. 2021; Fang et al. 2021), and social isolation resulting from lockdown measures (Ganesan et al. 2021; Fancourt et al. 2021; García‐Prado et al. 2022; Serrano‐Alarćon et al. 2022; Butterworth et al. 2022). The psychological consequences of the pandemic have been heterogeneously distributed across different demographic, socioeconomic, and occupational categories (Quintana‐Domeque and Proto 2022; Butterworth et al. 2022; Vloo et al. 2021; Amerio et al. 2021; Wilson et al. 2021; Kauhanen et al. 2023).

The literature closely related to our paper focuses on the impact of the pandemic on the mental health of different occupational groups. People who work in essential services, KWs, reports higher level of mental health deterioration than non‐KWs (Bu et al. 2022; Ayling et al. 2020; May et al. 2021). Disparities in mental health impact exist even among different KW categories (Toh et al. 2021; De Boni et al. 2020). This broader perspective on KWs sets the stage for a more focused examination of health and social care professionals, as they faced the highest levels of exposure to pandemic‐related risks. These professionals faced significant mental health challenges, including work‐related stressors such as high workload, inadequate resources, and emotional exhaustion (Bu et al. 2022; Duarte et al. 2022; Kapil et al. 2022; Lamb et al. 2022; Maddock 2024; Chireh et al. 2025).^2^ These challenges intensified during the COVID‐19 pandemic due to organizational shortcomings such as understaffing, unreliable access to personal protective equipment, and heightened fears of infecting others (Greene et al. 2021; Owens et al. 2024). However, evidence suggests that healthcare workers were managing their mental health relatively well compared to other KWs (Toh et al. 2021).

Among HSC workers, differences emerged across occupational groups. For example, among healthcare workers, nurses and other frontline staff show worsened psychological symptoms than other healthcare workers during the COVID‐19 pandemic (Lai et al. 2020; Vizheh et al. 2020; Molina et al. 2024; Maunder et al. 2024). Emotional distress of nurses and other frontline health workers are linked to hospital experiences of suffering and death, longer working hours, fear of COVID‐19 infection and spreading it to family members, lack of pro‐tective equipment, lack of social support and workplace discrimination (Dai et al. 2020; Yang et al. 2021; Khajuria et al. 2021). Complying with the general population trend, female and younger healthcare workers reported severe degrees of psychological symptoms compared to males and older (Lang et al. 2020; Zhu et al. 2020; Lai et al. 2020; Khajuria et al. 2021; Biber et al. 2022). Contrary to findings in population‐level studies, healthcare workers in areas with higher COVID‐19 infection rates reported higher mental health deterioration (Swaziek and Wozniak 2020; Vizheh et al. 2020).

Deteriorating mental health showed a direct link with the labor market during the pandemic, slowing down labor market activities. For example, sickness absences due to mental ill‐health rapidly increased in the English NHS at the beginning of the pandemic, with higher absences among healthcare workers aged 60 and above, and among ethnic minorities (van der Plaat et al. 2021). Healthcare staff viewed return to work as a health hazard, exacerbating their poor mental health (Tan et al. 2020). In turn, longer working hours also increased their psychological stress, exacerbating new and old psychological issues (Hino et al. 2022; Lang et al. 2020; Kotera et al. 2022; Søvold et al. 2021). The impact of these mental health challenges on the professional trajectories of healthcare workers was also a subject of concern, with a marked increase in turnover and attrition rates among these professionals and influencing their long‐term career decisions and long‐term plans (Mercado et al. 2022; Doleman et al. 2023; Guan et al. 2020).

Most of the above literature discussing the impact of the COVID‐19 pandemic on mental health on healthcare workforce relies on survey data collected during the pandemic or a comparison of outcome measures collected during the pandemic with their post‐pandemic period. While cross‐sectional studies provide valuable snapshots of the mental health status during the pandemic, they have limitations such as not accounting for pre‐pandemic mental health conditions or the longitudinal evolution of mental health symptoms. Longitudinal studies are more robust in understanding the differential changes and the progression of mental health issues over time. They help to establish a baseline and track changes, offering a clearer picture of the long‐term effects of the pandemic and across worker groups. To identify the long term impact on mental health, in this paper we exploit the longitudinal aspect of our data to explore trajectories in mental health, comparing data pre‐2020 to the period after the onset of the pandemic.

## 3 Data

We use data from the UK Household Longitudinal Study (UKHLS), also known as Understanding Society, a panel of individuals and their households which collects information about various aspects of their life, including health, employment and education. We draw on data from waves 7 to 13 of the main survey. Waves 7 to 10 constitute the pre‐pandemic period, with data collected prior to the onset of the pandemic. To ensure a cleaner pre‐ and post‐pandemic comparison, we only employ Wave 13 as the post‐treatment period. All interviews in wave 13 were conducted strictly after the pandemic began (planned fieldwork period for each UKHLS wave spans 24 months and data collection for wave 13 was carried out between January 2021 and May 2023). We purposely exclude waves 11 and 12 for which interviews were conducted during the COVID‐19 outbreak.^3^

We also exploit the information from the COVID‐19 waves collected by the UKHLS. In April 2020 respondents of the UKHLS main survey were invited to take part in the COVID‐19 study. The survey was intended to capture the implications of the pandemic on the welfare of individuals.^4^ Subsequent waves were collected in May, June, July, September and November 2020 and January, March and September 2021. We construct a dataset linking waves 7 to 10 from the main survey (covering the pre‐pandemic period) and the COVID‐19 surveys (to represent the post‐pandemic period). For the purpose of the paper, we use waves April to November 2020, and exclude the COVID‐19 surveys collected in 2021 to avoid an overlap with wave 13 collected for the main UKHLS.^5^

For the empirical analysis we therefore have two datasets (both sharing the same pre‐pandemic period), one using the main UKHLS waves (waves 7 to 10 & wave 13) and the second combining the main survey (waves 7–10) and the COVID‐19 surveys. For easiness of exposition we will refer to these as the main dataset and the COVID‐19 dataset, respectively. The COVID‐19 dataset allows us to estimate the effect of the pandemic in the short‐term while the main dataset captures the effects of the pandemic in the medium‐term. Where applicable, we present the results in chronological order, beginning with the analysis based on the COVID‐19 dataset, followed by the main survey dataset. However, for robustness checks and analytical extensions, we mainly rely on the main survey, as it includes more detailed information on a broader set of variables.

### 3.1 Mental Health

We use the General Health Questionnaire (GHQ) to capture changes in mental health. This widely used and validated instrument detects signs of psychological distress (Goldberg et al. 1997). The GHQ consists of 12 items assessing various aspects of mental well‐being, including concentration, sleep disturbances, sense of purpose, decision‐making ability, stress levels, difficulty overcoming problems, enjoyment of daily activities, ability to cope, feelings of depression, confidence levels, self‐worth, and overall happiness. The GHQ items are rated on a scale from 1 to 4, with higher scores indicating a worsening in the respondent's functioning or well‐being. Table A1 in the Supporting Information S1 lists the specific questions and response options.

To measure mental health changes, we employ four indicators. We first use the GHQ Caseness, which converts answers to the 12 questions recoding the scores of all items and converts them into a single score ranging from 0 (least distressed) to 12 (most distressed). Second, we use the GHQ Likert scale, which recodes responses on a scale from 0 to 3 (instead of the original 1–4) and sums all values across items, yielding a scale from 0 (least distressed) to 36 (most distressed). Additionally, we define two binary indicators that indicate whether the respondent is distressed, if the GHQ Likert score exceeds 15, and severely distressed, if it exceeds 20 (Ponizovsky et al. 2018).

**FIGURE 1:**
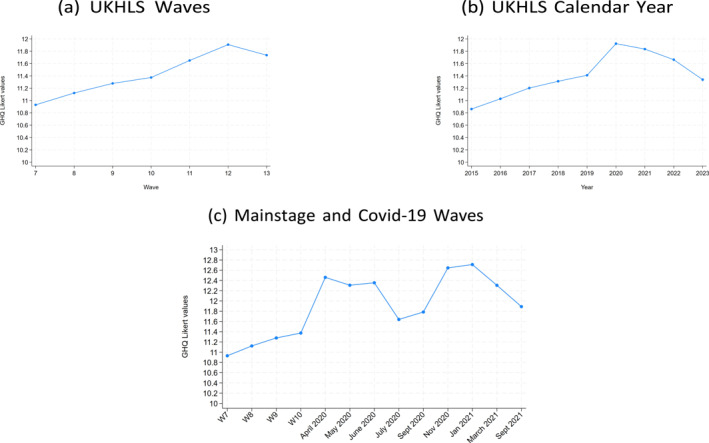
Trends in GHQ Likert. Graphs show trends in mental health (measured by the GHQ likert variable). Figure (a) shows the trends using data from wave 7 to wave 13. Figure (b) shows trends by calendar year, grouping respondents according to their interview year. Figure (c) shows trends in mental health using a sample that combines waves 7–10 (pre‐pandemic) with all COVID‐19 waves (post‐pandemic). Samples used in these figures include all respondents of the UKHLS (workers and non‐workers). Similar trends are observed when restricting the sample to those in employment.

Mental health was already deteriorating in the general population before the pandemic (Banks and Xu 2020). Using the UKHLS, we plot trends in mental health. Figure 1 shows the trends in the GHQ Likert measure. Figure 1a presents trends when using data from the main survey, waves 7 to 13. The figure indicates an overall worsening in mental health (note that higher scoring of the GHQ Likert reflects higher levels of distress), especially around waves 11 and 12 which overlap with the onset of the pandemic, and with a small recovery in wave 13. Figure 1b shows trends using data grouped according to calendar year of interview, as opposed to wave data, with a similar pattern of increasing deterioration in GHQ, especially in years 2020–2022, and a return to similar pre‐pandemic levels in 2023. Figure 1c shows the trends using the COVID‐19 dataset, that includes survey data spanning waves 7 to 10 for the pre‐pandemic period and all nine waves of the COVID‐19 survey waves.^6^ Trends in this figure show a decrease in mental health (reflected in an increase in the GHQ Likert measure) between April and July 2020 and between September 2020 and March 2021, coinciding with the first and second waves of the pandemic (and lockdowns) in the UK.

### 3.2 Worker Classification

For the purpose of this paper, we are mainly interested in the mental health of HSC workers. HSC workers represent a work category nested within the more general group of KWs. KWs are workers considered to provide services in critical sectors, and were officially defined at the start of the pandemic to determine the eligibility of children to attend their school in periods of lockdown (Department for Education 2020). The largest group of workers in key occupations were in HSC, but other occupations categorized as KWs also included workers in education and childcare, key public services, local and national government, food and other necessary goods, public safety and national security, transport, utilities and communication and financial services (ONS 2020). Our primary focus is on HSC KWs and throughout the paper we examine first their mental health relative to other KWs outside the HSC sector, followed by a comparison relative to non‐KWs. These group comparisons allow us to identify whether the mental health of HSC workers experienced worse outcomes compared to workers in other group classifications. To contextualize our results, we also perform a broader analysis between all KWs and non‐KWs.

In order to construct our variables of interest we follow the following steps. First, we identify KWs by cross‐tabulating industry and occupation classifications (using the 2010 Standard Occupational Classification (SOC) and the 2007 Standard Industry Classification (SIC)) available in the table published by the Office for National Statistics (Office for National Statistics 2020) listing those workers classified as KWs. Second, among those classified as KWs, we generate an indicator variable that takes value 1 for individuals employed in the HSC sector and 0 for all other essential workers (other KWs). Next, we construct an indicator that takes value 1 for HSC workers and 0 for workers in non‐essential occupations (non‐KWs).^7^ Table A2 in the Supporting Information S1 shows 46% of respondents in our sample are classed as KWs and 25% are HSC KWs. These figures are slightly above and below the percentage of KWs and HSC KWs, respectively, compared to national Figure^8^.

This detailed classification based on SIC and SOC codes is only available in the main surveys. The COVID‐19 surveys include only a sector‐based question: ”Are you working as a key worker in any of the key sectors below during the current coronavirus situation?” This question was asked in the May to September 2020 waves. Given the temporal proximity of the April and May 2020 surveys, we impute the April 2020 KW status using information collected in May 2020. Based on this information, we classify respondents as KWs (subdivided into HSC workers and other KWs) and non‐KWs, following the same steps outlined above.^9^

**FIGURE 2:**
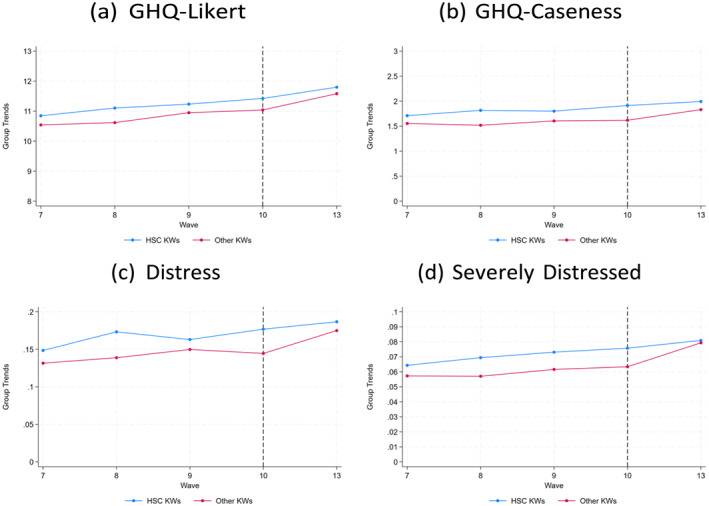
Trends in mental health for HSC KWs versus other KWs. Figures show trends for all measures of mental health examined in the empirical analysis for the treatment group HSC KWs and control group other KWs. Sample employed to generate the figures comprises KWs participating in waves 7 to 10 (pre‐pandemic) and wave 13 (post‐pandemic). The vertical dotted line indicates the wave prior to the onset of the pandemic.

**FIGURE 3:**
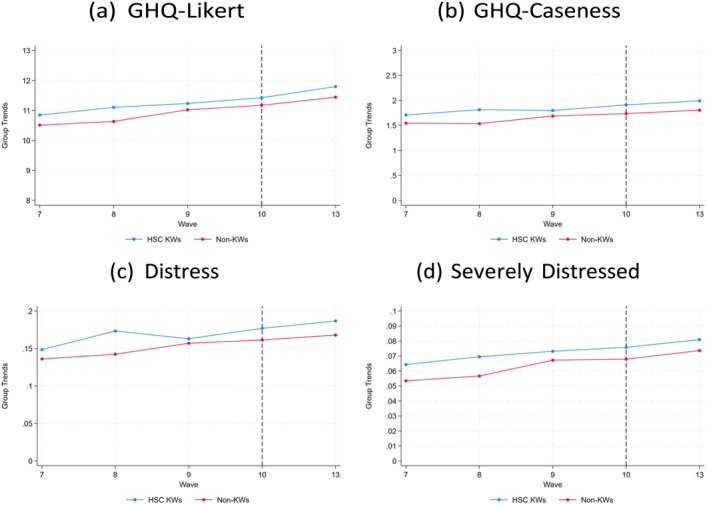
Trends in Mental Health for HSCWs versus Non‐KWs. Figures show trends for all measures of mental health examined in the empirical analysis for the treatment group HSC KWs and control group non‐KWs. Sample employed to generate the figures comprises KWs participating in waves 7 to 10 (pre‐pandemic) and wave 13 (post‐pandemic). The vertical dotted line indicates the wave prior to the onset of the pandemic.

Figures 2 and 3 show, using data from the mains surveys, the trends in mental health (for all four measures studied) before and after the onset of the pandemic comparing HSC workers to other KWs and non‐KWs, respectively. A similar pattern emerges across graphs. Whereas the GHQ Likert and Caseness variables are very similar for the comparison groups, there is a bigger gap in trends between groups when we examine the distressed and severely distressed mental health indicator variables. All graphs display a similar stylized fact, in that HSC KWs have worse mental health than other KWs and non‐KWs and the gap between groups is not markedly different after the start of the pandemic. Similar patterns are observed when using data from the COVID‐19 surveys, as shown in Figures A2 and A3 in the Supporting Information S1.

### 3.3 Control Variables

The estimation strategy incorporates several control variables to account for any residual differences between HSC KWs and the comparator worker groups. We include a set of variables not influenced by the pandemic. Specifically, our set of controls include age, education, marital status, number of children in the household, household income, year of interview and regional dummies. These variables jointly account for baseline differences in socioeconomic position, family structure, temporal and geographic context that may correlate with both occupational sector and mental health trajectories, thereby improving the plausibility of conditional parallel trends and mitigating bias from time‐varying confounding. We use a fixed effects estimator in our empirical strategy, and hence exclude time‐constant variables such as gender and ethnicity that would be dropped in the within transformation. However, gender and ethnicity are variables that potentially determine mental health. To investigate this potential heterogeneity in treatment effects, we also examine differences by gender and ethnic subgroups and briefly discuss the results below.

Descriptive statistics for both the mental health and control variables for the main dataset and the COVID‐19 dataset are presented in Tables A2 and A3 in the Supporting Information S1. These tables report the mean and standard deviation before the onset of the pandemic (waves 7–10) and post‐pandemic, wave 13 for the main sample and waves April to November 2020 for the COVID‐19 sample.

## 4 Empirical Strategy

### 4.1 Baseline Difference‐in‐Differences Design

To examine whether HSC workers experienced distinct mental health trajectories relative to other occupational groups, we apply a difference‐in‐differences (DiD) model. In the canonical DiD setup, treatment status varies both across groups and over time: one group remains untreated throughout, while the other is exposed only after a baseline period. Let *Y*_*it*_ denote the mental health outcome for individual *i* observed in time *t*. Let *G*_*i*_ be an indicator variable identifying worker classification of individual *i*, where *G* ∈ {0, 1}, with *G* = 1 denoting HSC workers and *G* = 0 the comparison group. Define *Post*_*t≥*2020_ ∈ {0, 1} as an indicator for the pre and post‐pandemic period, with value *Post*_*t≥*2020_ = 0 in the pre‐pandemic period and *Post*_*t≥*2020_ = 1 after the onset of the pandemic. In a standard two‐way fixed‐effects framework, the model is defined as follows:

(1) $$Y_{it}=\beta_{0}+\beta_{1}Post_{t\geq 2020}+\beta_{2}G_{i}+\beta_{3}\left(G_{i}\times Post_{t\geq 2020}\right)+\alpha_{i}+\delta_{t}+\varepsilon_{it}$$

where *β*_3_ is the average treatment effect on the HSC group under the assumption of parallel trends. This assumption states that, in the absence of the pandemic, average outcomes for HSC workers and control worker groups would have followed the same trajectory over time. *α*_*i*_ captures individual fixed‐effects, *δ*_*t*_ denotes time fixed‐effects and *ε*_*it*_ is the error term.

Our empirical setting deviates from this canonical case. The COVID‐19 pandemic constitutes a universal shock: all workers are exposed to the pandemic in *t* = 1. Following Shahn and Hatfield (2024), our design is best viewed as a pre–post study with heterogeneous groups, where the interaction term *G*_*i*_ × *Post*_*t*_ captures differences in the impact of the pandemic across groups, rather than the absolute effect within any single group. Under this interpretation, the DiD estimator can be written as

(2) $$\beta_{3}=E\left[Y_{i1}-Y_{i0}|G=1\right]-E\left[Y_{i1}-Y_{i0}|G=0\right]$$

that is, the difference in average changes in mental health outcomes between HSC workers and the comparison group. In potential‐outcomes notation, identification relies on a group‐specific parallel trends assumption:

(3) $$E\left[Y_{i1}\left(0\right)-Y_{i0}\left(0\right)|G=1\right]=E\left[Y_{i1}\left(0\right)-Y_{i0}\left(0\right)|G=0\right].$$

This assumption implies that, absent COVID‐19, changes in mental health between *t* = 0 and *t* = 1 would have been the same across occupational groups, so that deviations from this pattern can be attributed to heterogeneous effects of the pandemic. A second identifying assumption is that of no anticipation. Given the sudden and unexpected onset of COVID‐19, we consider anticipatory effects unlikely.

### 4.2 Conditional Parallel Trends and Covariate Adjustment

Visual inspection of the raw pre‐pandemic trends (in Figures 2 and 3) suggests that unconditional parallel trends may not be fully plausible. To account for potential differences in observed characteristics that could drive differential trends, we therefore adopt a conditional parallel trends assumption:

(4) $$E\left[Y_{i1}\left(0\right)-Y_{i0}\left(0\right)\;\left|\;G=1,X]=E[Y_{i1}\left(0\right)-Y_{i0}\left(0\right)\;\right|\;G=0,X\right],$$

where *X*_*it*_ is a vector of observed characteristics. Under this assumption, mental health trajectories across groups would have evolved similarly in the absence of the pandemic, conditional on *X*. We implement this by estimating the following two‐way fixed‐effects model with covariates:

(5) $$Y_{\mathrm{it}}=\beta_{0}+\beta_{1}{\text{Post}}_{t\geq 2020}+\beta_{2}G_{i}+\beta_{3}\left(G_{i}\times {\text{Post}}_{t\geq 2020}\right)+\beta_{4}X_{\mathrm{it}}+\alpha_{i}+\delta_{t}+\varepsilon_{\mathrm{it}},$$

where *X*_*it*_ includes socioeconomic characteristics, household structure, and regional indicators known to influence both mental health and occupational sorting. In this specification, *β*_3_ is interpreted as the difference in average mental health changes between HSC workers and the comparison group, conditional on *X*. All DiD specifications are estimated using a fixed effects model with clustered standard errors at the household level.

### 4.3 Dynamic Specification and Assessment of Identifying Assumptions

While the conditional parallel trends assumption is not directly testable, it can be examined using a dynamic specification that traces group‐specific trajectories over multiple periods before and after the onset of the pandemic. To this end, we estimate an event‐study‐type model that replaces the single *Post*_*t*_ indicator with a full set of time dummies interacted with group status:

(6) $$Y_{it}=\beta_{0}+\sum_{\tau \;/=\tau_{0}}\gamma_{\tau }\;1\left\{t=\tau \;\right\}+\sum_{\tau \;/=\tau_{0}}\theta_{\tau }\;G_{i}\times 1\left\{t=\tau \;\right\}+\beta_{4}X_{it}+\alpha_{i}+\delta_{t}+\varepsilon_{it}$$

where *α*_*i*_ denotes individual fixed effects, *τ* indexes survey waves, and *τ*_0_ is a chosen pre‐pandemic reference period. The coefficients *θ*_*τ*_ trace out the evolution of mental health differences between HSC workers and the comparison group relative to *τ*_0_.

This dynamic specification serves two purposes. First, the pattern of pre‐pandemic coefficients *θ*_*τ*_ (for *τ* *<* *τ*_0_) provides evidence on whether group‐specific trends were approximately parallel before COVID‐19, once covariates are controlled for. Second, the post‐pandemic coefficients *θ*_*τ*_ (for *τ* ≥ *τ*_0_) describe the timing and persistence of heterogeneous effects across groups.

### 4.4 Alternative Estimators Under Conditional Independence in Past Outcomes

Even if conditional parallel trends hold approximately, it is useful to consider alternative identification strategies that relax this assumption and instead rely on conditional independence given past outcomes, as proposed by O'Neill et al. (2016). Our data include several pre‐pandemic waves of the GHQ, which we exploit in two complementary ways. First, we estimate the following lagged dependent variable (LDV) model

(7) $$Y_{i}=\beta_{0}+{\sum_{\tau =7}^{10}\gamma }_{\tau }\;Y_{i\tau }+\beta_{1}G_{i}+\beta_{2}X_{it}+\alpha_{i}+\delta_{t}+\varepsilon_{it}$$

where *Y*_*i*_, the post‐pandemic mental health in wave 13, is regressed on pre‐pandemic mental health outcomes *Y*_*iτ*_ for waves *τ* = 7, 8, 9, 10, group status *G*_*i*_, and a set of covariates *X*_*it*_. This approach does not require parallel trends but instead assumes that observable pre‐pandemic outcomes and covariates are rich enough to proxy for unobserved determinants of mental health trajectories. *G*_*i*_ captures differences between the HSC group and comparator groups not explained by past mental health as represented by the coefficient *β*_1_.

Second, we implement a matching plus DID approach. We match HSC workers to other workers with similar observed characteristics pre‐pandemic. Units are matched using a one‐to‐one nearest neighbor matching process without replacement.^10^ We next estimate the DiD model depicted in Equation (5) on the matched sample. The subsequent DiD on this matched sample then compares changes in outcomes between groups that were already highly comparable before the pandemic. This approach again relaxes the need for parallel trends in the full sample, as it enforces balance in pre‐pandemic outcomes and observables by construction.

### 4.5 Attrition and Inverse Probability Weighting

Finally, all our specifications correct for potential bias due to survey attrition. Individuals may leave and re‐enter the sample across waves, and attrition might be correlated with both mental health and occupational status. We address this by constructing inverse probability weights (IPWs) that reweight the observed sample to resemble the original population.

Specifically, we estimate at each wave the probability of response as a function of a rich set of covariates measured at the initial wave (wave 7), including gender, age, ethnicity, education, marital status, household size, household income, employment status, physical and mental health as captured by the SF‐12 questionnaire, and regional dummies. The IPW for each respondent is defined as the inverse of the estimated response probability. All DiD, dynamic, LDV and matching‐based estimators are then estimated using these weights. Under the assumption of conditional independence of response, this weighting procedure mitigates biases arising from selective attrition.

## 5 Results

We start with an exploratory analysis on the overall association between the pandemic and population mental health. We run a simple regression of each mental health variable on a dummy reflecting the post‐pandemic period. Table A4 in the Supporting Information S1 shows the estimates obtained. The top panel presents the results when using the COVID‐19 sample and the bottom panel the estimates obtained using the sample from the main waves. Overall coefficients are positive and statistically significant, indicating that the pandemic has a strong association with worsened mental health. While examining the overall association between the pandemic and mental health outcomes represents a valid preliminary step, in subsequent analyses we move beyond aggregate effects to explore heterogeneous effects across worker groups. In particular, we now examine heterogeneity in effects by comparing outcomes between HSC KWs and other worker groups: other KWs and non‐KWs. In sections below we first examine the effects across groups in the short‐term using the COVID‐19 waves and the effects in the medium‐term using the main waves. We also present some robustness tests, extensions and potential mechanisms that explain our results.

### 5.1 The Effect of the Pandemic in the Short‐Term

We examine the relative change that the COVID‐19 pandemic has brought about on the mental health of HSC KWs by using the sample that captures the experiences of respondents in the months immediately after the onset of the pandemic. For this purpose we use the COVID‐19 dataset to estimate the short‐term effects of the pandemic waves between April and November 2020. Table 1 presents the estimates from the DID model specified in Equation (5) for each of the four mental health variables used, all based on the GHQ questionnaire: the GHQ Likert and GHQ Caseness variables, and the indicators variables for distress and severely distressed. Each panel corresponds to a different comparison group: the top panel compares HSC KWs with other KWs (KWs in non‐HSC occupations) and the bottom panel compares HSC KWs with non‐KWs. Overall, results in Table 1 suggest that, using conventional significance levels, there are no differences in mental health between HSC KWs and any of the comparator worker groups, indicating mental health for the HSC group deteriorates in line with deterioration of other worker groups. At 10% significance level, there is only some indication that mental health is worsened for HSC KW compared to other KWs. We replicate the analysis on a balanced sample of respondents present in all waves before (waves 7 to 10 of the main survey) and after the pandemic (waves April, May, June, July, September and November 2020). Results are available in Table A5 in the Supporting Information S1 and are similar to the main results, suggesting the estimates are not sensitive to sample composition.^11^

**TABLE 1:** Effects of the pandemic on mental health ‐ short‐term effects.

	(1) GHQ likert	(2) GHQ caseness	(3) Distress	(4) Severe distress
HSC KWs versus other KWs	0.316^+^	0.151	0.023^+^	0.004
	(0.176)	(0.105)	(0.014)	(0.009)
*R* ^2^	0.626	0.574	0.509	0.462
NT	35,688	35,688	35,688	35,688
*N*	8694	8694	8694	8694
HSC KWs versus Non‐KWs	−0.094	−0.120	−0.011	−0.002
	(0.162)	(0.097)	(0.013)	(0.009)
*R* ^2^	0.624	0.573	0.502	0.477
NT	50,180	50,180	50,180	50,180
*N*	12,135	12,135	12,135	12,135

*Note:* Sample includes waves 7–10 (pre‐pandemic) and waves April to November 2020 (post‐pandemic). All model specifications incorporate IPWs to adjust for attrition. Controls included are age, education, marital status, number of children aged 0–15, household income, region dummies and interview year dummies. Estimates obtained using the unbalanced sample. Standard errors clustered at the household level. NT is the total number of observations and *N* is number of individual respondents in the sample.

Significance levels: ^+^ *p <* 0.10, **p < 0.05, ***p < 0.01.

The estimates shown in Table 1 present the average effect over the April to November 2020 period. To assess potential time‐varying effects, we estimate event‐type models. Results are presented in Figures A5 and A6 in the Supporting Information S1. These figures are useful to check the behavior of the estimates in the pre‐treatment period, and the statistically insignificant estimates pre‐pandemic suggest the parallel trends assumptions holds. With the exception of the estimates across several mental health measures in April 2020 for HSC KWs relative to other KWs, all other estimates are statistically insignificant. This suggests that there is a deterioration in mental health for this group immediately after the start of the pandemic but this effect dissipates in subsequent periods.

### 5.2 Medium‐Term Effects of the Pandemic

We next examine the effect of the pandemic in the medium‐term using the main survey dataset. Table 2 presents the results obtained from the DID model described in Equation (5) comparing occupational groups. As in Table 1, we present the results for all four mental health outcomes studied and each panel shows the estimates corresponding to the comparison between the HSC KW group and relevant occupational groups. All point estimates are statistically insignificant, suggesting there is no deterioration in the mental health of HSC KWs. We also re‐estimate the model in Table 2 using the balanced panel sample consisting of individuals present in all relevant waves (waves 7–13). Table A6 in the Supporting Information S1 presents the estimates, indicating that our results remain consistent with those obtained from the full sample. In line with the analysis for the short‐term impact of the pandemic, we also run event‐type heterogeneity analysis. This is available in Figures A7 and A8 in the Supporting Information S1. Estimates for the pre‐pandemic period suggest there are no pre‐trends, in line with the results from the short‐term analysis. Given there is only a single post‐treatment period, these graphical representations are not informative of post‐treatment heterogeneity and estimates shown align closely with those reported in Table 1.

**TABLE 2:** Effects of the pandemic on mental health ‐ medium‐term effects.

	(1) GHQ likert	(2) GHQ caseness	(3) Distress	(4) Severe distress
HSC KWs versus other KW	−0.051	−0.057	−0.014	−0.001
	(0.225)	(0.137)	(0.017)	(0.013)
*R* ^2^	0.620	0.571	0.522	0.471
NT	23,043	23,043	23,043	23,043
*N*	6999	6999	6999	6999
HSC KWs versus non‐KW	0.165	0.131	0.017	0.021^+^
	(0.212)	(0.130)	(0.016)	(0.012)
*R* ^2^	0.622	0.570	0.519	0.471
NT	32,552	32,552	32,552	32,552
*N*	10,013	10,013	10,013	10,013

*Note:* Sample includes waves 7–10 (pre‐pandemic) and wave 13 (post‐pandemic). All model specifications incorporate IPWs to adjust for attrition. Controls included are age, education, marital status, number of children aged 0–15, household income, region dummies and interview year dummies. Es‐timates obtained using the unbalanced sample. Standard errors clustered at the household level. NT is the total number of observations and *N* is number of individual respondents in the sample.

Significance levels: ^+^ *p <* 0.10, **p < 0.05, ***p < 0.01.

Estimates presented in Table 2 rely on the plausibility of the parallel trends assumption. Graphical analysis and regression estimates for the pre‐pandemic period provide support for this assumption. However, we complement the DID estimates presented above with an alternative identifying assumption that relies on the independence of outcomes conditional on past outcomes. Following O'Neill et al. (2016), we first employ the LDV approach where mental health outcomes in the post‐treatment period (wave 13) are regressed on mental health outcomes in waves 7 to 10 and a dummy for our treatment group HSC KWs. The estimated coefficient for the treatment group reflects the differential in mental health across worker groups. Results of this approach are presented in Supporting Information S1 Table A7. In line with our main set of results, we find no evidence of differences in mental health between HSC workers and other worker groups. The second approach, first matches treated and control units based on pre‐treatment observed characteristics followed by a DID model on the matched sample. Estimates are presented in Table A8 in the Supporting Information S1 and confirm the results obtained using the LDV approach of no mental health differences across groups.

The results for the medium‐term effects are based on a sample that exploits suitable waves pre and post‐pandemic. We next move to do the analysis based on the calendar year of interview, rather than based on survey waves. By focusing on interview year, the analysis accounts for year‐to‐year changes in mental health, potentially enabling a more precise examination of temporal shifts in data. This is particularly useful to reflect actual changes occurring during and after the pandemic rather than determined by a predefined grouping of waves. For this analysis we use information from wave 7 to wave 13 (including waves 11 and 12) and construct a dataset based on the calendar year of interview.^12^ We apply the DID estimator and the corresponding results are presented in Table 3. The estimates show no statistically significant differences between HSC KWs and the other two groups. For further insight on the potential heterogeneity of the estimates post‐pandemic, for each comparison group we plot the estimates for each time period. We include these in Figures A9 and A10 in the Supporting Information S1. The figures suggest there is no discernible heterogeneity in effects across years post‐pandemic. Overall, the results obtained using calendar yearly data are in line with those obtained using wave information.

**TABLE 3:** Effects of the pandemic on mental health ‐ medium‐term effects using calendar year data.

	(1) GHQ likert	(2) GHQ caseness	(3) Distress	(4) Severe distress
HSC KWs versus other KWs	−0.032	−0.089	−0.004	−0.010
	(0.140)	(0.085)	(0.011)	(0.008)
*R* ^2^	0.593	0.535	0.478	0.433
NT	32,350	32,350	32,350	32,350
*N*	8088	8088	8088	8088
HSC KWs versus non‐KWs	0.051	−0.012	0.005	−0.000
	(0.134)	(0.082)	(0.011)	(0.008)
*R* ^2^	0.592	0.537	0.479	0.433
NT	45,771	45,771	45,771	45,771
*N*	11,629	11,629	11,629	11,629

*Note:* Sample constructed based on Waves 7–13 (including waves 11 and 12) and grouped by calendar year of interviewed, from 2015 to 2022. Waves 11 and 12 are included and the resulting sample size is larger than in Table 2. All model specifications incorporate IPWs to adjust for attrition. Controls included are age, education, marital status, number of children aged 0–15, household income, region dummies and interview year dummies. Estimates obtained using the unbalanced sample. Standard errors clustered at the household level. NT is the total number of observations and *N* is number of individual respondents in the sample.

Significance levels: ^+^ *p <* 0.10, **p < 0.05, ***p < 0.01.

### 5.3 Robustness Checks and Extensions

To assess the robustness of our results, we conduct some checks, all based on the main sample. First, we limit the sample to individuals who are continuously employed across waves to reduce potential bias arising from employment transitions. Changes in employment status could potentially improve mental health outcomes, reducing the ability to isolate changes in mental health induced by the pandemic. Table 4 presents the results. Among those who remained in the same job, we find no evidence of worsened mental health for HSC workers, with the exception of an increase in the likelihood of being severely distressed compared to non‐KWs, only statistically significant at 10% level.

**TABLE 4:** Respondents continuously employed.

	(1) GHQ likert	(2) GHQ caseness	(3) Distress	(4) Severely distressed
HSC KWs versus other KWs	−0.081	−0.035	−0.032	−0.002
	(0.304)	(0.185)	(0.023)	(0.017)
*R* ^2^	0.607	0.552	0.507	0.437
NT	11,000	11,000	11,000	11,000
*N*	3085	3085	3085	3085
HSC KWs versus non‐KWs	0.298	0.212	0.008	0.031^+^
	(0.298)	(0.181)	(0.023)	(0.017)
*R* ^2^	0.611	0.554	0.498	0.443
NT	12,589	12,589	12,589	12,589
*N*	3536	3536	3536	3536

*Note:* Sample includes waves 7–10 (pre‐pandemic) and wave 13 (post‐pandemic). Sample restricted to those individuals that were continuously employed between Wave 7 and 13. All model specifications incorporate IPWs to adjust for attrition. Controls included are age, education, marital status, number of children aged 0–15, household income, region dummies and interview year dummies. Estimates obtained using the unbalanced sample. Standard errors clustered at the household level. NT is the total number of observations and *N* is number of individual respondents in the sample.

Significance levels:^+^ *p <* 0.10, **p < 0.05, ***p < 0.01.

Second, to mitigate potential bias from pre‐existing mental health issues, we restrict the sample to individuals that prior to 2020 reported good mental health, defined as those not distressed (having a GHQ Likert lower than 15). Some individuals may have experienced poor mental health prior to the onset of COVID‐19, either as a condition independent of the pandemic or as an early deterioration influenced by pandemic stressors. By focusing on workers with a healthy pre‐pandemic baseline, we aim to capture pandemic‐induced changes in mental health, strengthening the validity of our estimates within our identification strategy. Table 5 presents the results from the model using this restricted sample. HSC workers show increased probability of distress or severe distress in relation to non‐KWs. This is only statistically significant at 10% level. All other estimates indicate there is no difference in mental health of HSC workers compared to other worker groups. We also perform a robustness check restricting the sample to those without severe distress prior to 2020 (but who may have been mildly distressed). The results, shown in Supporting Information S1 Table A9, indicate no significant effects following the onset of the pandemic.

**TABLE 5:** Respondents with no psychological distress pre‐pandemic.

	(1) GHQ likert	(2) GHQ caseness	(3) Distress	(4) Severe distress
HSC KWs versus other KWs	−0.015	−0.041	−0.006	0.007
	(0.222)	(0.127)	(0.015)	(0.010)
*R* ^2^	0.570	0.418	0.364	0.301
NT	15,680	15,680	15,680	15,680
*N*	4736	4736	4736	4736
HSC KWs versus non‐KWs	0.122	0.130	0.024^+^	0.017^+^
	(0.209)	(0.118)	(0.014)	(0.010)
*R* ^2^	0.568	0.428	0.356	0.299
NT	21,669	21,669	21,669	21,669
*N*	6668	6668	6668	6668

*Note:* Sample includes waves 7–10 (pre‐pandemic) and wave 13 (post‐pandemic). Sample restricted to those individuals with no distress (GHQ *>* 15) in waves 7–10 prior to the pandemic. All model specifications incorporate IPWs to adjust for attrition. Controls included are age, education, marital status, number of children aged 0–15, household income, region dummies and interview year dummies. Estimates obtained using the unbalanced sample. Standard errors clustered at the household level. NT is the total number of observations and *N* is number of individual respondents in the sample.

Significance levels: ^+^ *p <* 0.10, **p < 0.05, ***p < 0.01.

Third, we consider whether differences in mental health could arise due to differences across HSC workers on exposure levels to the pandemic. Among HSC KWs, frontline workers experienced higher workload than average and increased stress due to uncertainty in working conditions and consequently reported higher mental health distress (van der Goot et al. 2021). With the purpose to better understand the impact on HSC KWs, we first identify workers in the frontline among HSC KWs.^13^ We then divide frontline health care (HC) from frontline social care (SC) workers. Each of these frontline groups is compared to other KWs and non‐ KWs. Finally, we compare frontline HC and SC workers. All results of these estimations are available in Table 6. We find no differences among any of these groups, except for the case of frontline HC, that show an increase in the probability of being distressed when compared to non‐KWs. It is worth noting that restricting the analysis to these detailed subgroup classifications decreases the sample size, potentially driving the lack of findings.

**TABLE 6:** Differences in mental health for frontline workers.

	(1) GHQ likert	(2) GHQ caseness	(3) Distress	(4) Severely distressed
Frontline HSC versus other KWs	−0.116	−0.076	−0.014	−0.003
	(0.310)	(0.190)	(0.024)	(0.018)
_*R*_^2^	0.621	0.573	0.524	0.470
NT	20,306	20,306	20,306	20,306
*N*	6237	6237	6237	6237
Frontline HSC versus non‐KWs	−0.085	0.007	0.011	0.007
	(0.297)	(0.184)	(0.023)	(0.018)
_*R*_2	0.624	0.572	0.521	0.473
NT	29,747	29,747	29,747	29,747
*N*	9236	9236	9236	9236
Frontline HC versus other KWs	−0.035	0.152	0.036	−0.008
	(0.521)	(0.339)	(0.044)	(0.030)
_*R*_^2^	0.621	0.571	0.522	0.468
NT	18,455	18,455	18,455	18,455
*N*	5660	5660	5660	5660
Frontline HC versus non‐KWs	0.178	0.306	0.078^+^	−0.001
	(0.523)	(0.339)	(0.044)	(0.030)
_*R*_^2^	0.623	0.571	0.520	0.473
NT	27,863	27,863	27,863	27,863
*N*	8658	8658	8658	8658
Frontline SC versus other KWs	0.020	−0.089	−0.025	0.003
	(0.387)	(0.231)	(0.028)	(0.023)
_*R*_^2^	0.622	0.574	0.526	0.469
NT	18,957	18,957	18,957	18,957
*N*	5844	5844	5844	5844
Frontline SC versus non‐KWs	−0.101	−0.065	−0.010	0.016
	(0.370)	(0.226)	(0.028)	(0.023)
_*R*_^2^	0.625	0.573	0.522	0.473
NT	28,394	28,394	28,394	28,394
*N*	8843	8843	8843	8843
Frontline HC versus SC	0.529	0.432	0.081	−0.003
	(0.654)	(0.425)	(0.053)	(0.041)
_*R*_^2^	0.657	0.613	0.556	0.510
NT	2898	2898	2898	2898
*N*	930	930	930	930

*Note:* HC denotes healthcare KWs and SC denotes social care KWs. Sample includes waves 7–10 (pre‐pandemic) and wave 13 (post‐pandemic). All model specifications incorporate IPWs to adjust for attrition. Controls included are age, education, marital status, number of children aged 0–15, household income, region dummies and interview year dummies. Estimates obtained using the unbalanced sample. Standard errors clustered at the household level. NT is the total number of observations and *N* is number of individual respondents in the sample.

Significance levels: ^+^ *p <* 0.10, **p < 0.05, ***p < 0.01.

Fourth, we perform additional analysis to assess the relative change of the pandemic across demographic groups. This analysis is motivated by the fact that our fixed effects model excludes time‐invariant characteristics such as gender and ethnicity through the within transformation. To investigate potential heterogeneity in the estimated coefficients along these dimensions, we run subgroup analyses by estimating the model separately for subsamples defined by gender and ethnicity (ethnic minority and white background). Tables A10 and A11 in the Supporting Information S1 show the estimates obtained. Results suggest women in the HSC KW group are more likely to suffer severe distress compared to non‐KWs. The analysis by ethnic group indicates that HSC KWs of ethnic minority are more likely to experience severe distress compared to other KWs and non‐KWs and report worse health via the GHQ Likert measure. Most of these results are only statistically significant at 10% level.

Finally, we examine the individual questions of the GHQ. The GHQ questionnaire consists of 12 items, each with four possible responses. Based on these questions, we create indicator variables if the respondent marks a positive answer to the question.^14^ Table A12 in the Supporting Information S1 presents the results. HSC KWs reported a reduction in feelings of worthlessness, potentially reflecting the public narrative that individuals in the HSC sector played a key role in healthcare service delivery. By contrast, HSC KWs are more likely to feel constantly under strain compared to non‐KWs.

### 5.4 Alternative Measures of Mental Health

The analysis presented above employs measures of mental health based on the GHQ. We next use alternative measures of mental health outcomes to assess the sensitivity of our findings to the choice of outcome measures. In particular, we draw on the short form survey SF‐12, a self‐reported measure on the health of individuals. The SF‐12 survey is a 12‐item questionnaire used to generate scores for the physical component summary (PCS) and the mental component summary (MCS) (Ware et al. 1996). We use the MCS component of the SF‐12, only available across all main survey waves.^15^ The SF‐12 MCS score reflects an individual's mental health status, well‐being, and emotional functioning. It converts answers of the questionnaire into a single score for mental health ranging between 0 and 100 (from low to high functioning). In addition, we examine a subset of individual SF‐12 components, focusing on specific questions related to mental health experiences: whether they accomplished less, worked less carefully, whether they felt calm, they felt depressed, and whether their emotional problems interfered with social activities.^16^

Table 7 presents the results of the specification using the SF‐12 variables. The estimates for the overall MCS score are statistically insignificant, in line with the results of specifications using the GHQ variable. However, a closer examination across the individual components of the MCS reveals some patterns. HSC KWs are less likely to report feeling depressed compared to other KWs. Additionally, HSC KWs are less likely to feel they accomplished less or worked less carefully relative to non‐KWs.

**TABLE 7:** Alternative measures of mental health.

	(1)	(2)	(3)	(4)	(5)	(6)
	SF‐12 MCS	Accomplished less	Worked less carefully	Felt calm and peaceful	Felt downhearted and depressed	Health altered social life
HSC KWs versus other KWs	1.944	−0.065	−0.041	0.004	−0.106^**^	0.044
	(1.706)	(0.048)	(0.036)	(0.099)	(0.052)	(0.045)
*R* ^2^	0.662	0.454	0.433	0.573	0.474	0.478
NT	23,804	23,992	23,980	24,019	24,009	24,015
*N*	7300	7352	7349	7357	7355	7354
HSC KWs versus non‐KWs	−0.346	−0.171^***^	−0.123^***^	−0.028	−0.079	−0.019
	(1.601)	(0.042)	(0.036)	(0.084)	(0.052)	(0.049)
*R* ^2^	0.665	0.471	0.454	0.576	0.472	0.463
NT	33,927	34,239	34,199	34,258	34,240	34,265
*N*	10,554	10,634	10,619	10,640	10,635	10,643

*Note:* All model specifications incorporate IPWs to adjust for attrition. Controls included are age, education, marital status, number of children aged 0 to 15, household income, region dummies and interview year dummies. Estimates obtained using the unbalanced sample. Standard errors clustered at the household level. NT is the total number of observations and *N* is number of individual respondents in the sample.

Significant Level: ^+^ *p <* 0.10, **p < 0.05, ***p < 0.01.

### 5.5 Possible Mechanisms

Contrary to widely held assumptions that HSC KWs experienced a distinctively severe impact during the pandemic, our analysis finds no discernible effects of a significantly greater mental health decline in this group compared to others. This should not be interpreted as an absence of deterioration but to a decline in mental health that was broadly similar to those observed across the labor force. These findings prompt further examination into the underlying channels that may explain our findings.

Two of the main channels for worsened mental health during the pandemic relate to worries on income insecurity and social isolation (Fang et al. 2021; Foremny et al. 2024; Ganesan et al. 2021; Fancourt et al. 2021). Drawing on this evidence, our discussion of potential mechanisms focuses on the role of these two factors. The UKHLS contains variables capturing both dimensions, including respondents' subjective valuation of their financial stability in the present and future, and indicators of social interactions based on self‐reported levels of loneliness and isolation. We generate four indicator variables reflecting a positive assessment of their financial position and social connectedness.^17^ We estimate a DID model specified as in Equation (5) above, where the dependent variable *Y*_*it*_ now refers to one of the financial or social interconnectedness variables examined.

We follow the same chronology as in Sections 5.1 and 5.2, first examining these potential mechanisms in the short‐ and medium‐term. The short‐term analysis includes subjective measures of financial stability and information on loneliness, but not on isolation which was not collected in the COVID‐19 surveys.^18^ Results are presented in Table 8. While there is no difference between HSC KWs and other KWs, the findings suggest that relative to non‐ KWs, HSC KWs experience better current and future financial stability and no difference in loneliness. An examination of heterogeneous temporal effects, shown in Figures A11 and A12 in the Supporting Information S1, suggest that when present, these effects are more pronounced in periods immediately after the onset of the pandemic and subsequently become negligible.

**TABLE 8:** Mechanisms ‐ short‐term analysis.

	(1)	(2)	(3)
	Current financial situation	Future financial situation	Loneliness
HSC KWs versus other KW	0.010	−0.007	−0.016
	(0.014)	(0.015)	(0.020)
*R* ^2^	0.655	0.452	0.640
NT	31,167	24,985	23,982
*N*	8631	7787	6484
HSC KWs versus non‐KWs	0.054^***^	0.049^***^	0.009
	(0.013)	(0.014)	(0.018)
*R* ^2^	0.646	0.447	0.641
NT	43,946	35,274	33,888
*N*	12,083	11,061	8956

*Note:* Sample includes waves 7–10 (pre‐pandemic) and waves April to November 2020 (post‐pandemic). Outcome variable on current financial stability is available in waves April, May, July and November 2020. Outcome variable on future financial stability is available for May and July 2020. Outcome variable on loneliness is available for all wave between April and November 2020. All model specifications incorporate IPWs to adjust for attrition. Controls included are age, education, marital status, number of children aged 0–15, household income, region dummies and interview year dummies. Estimates obtained using the unbalanced sample. Standard errors clustered at the household level. NT is the total number of observations and *N* is number of individual respondents in the sample.

Significance levels: ^+^ *p <* 0.10, **p < 0.05, ***p < 0.01.

The analysis for the medium‐term, shown in Table 9, indicates no differences across groups in subjective financial stability or their loneliness/isolation indicators. We further check these results using the sample grouped by calendar year of interview. Results are available in Table A13 in the Supporting Information S1. This analysis reveals effects not detected using wave information. There is evidence of better subjective valuation of current finances and social interactions of HSC KWs relative to other worker groups.

**TABLE 9:** Mechanisms ‐ medium‐term analysis.

	(1)	(2)	(3)	(4)
	Current financial situation	Future financial situation	Loneliness	Isolation
HSC KWs versus other KWs	0.029	−0.006	0.010	0.026
	(0.018)	(0.018)	(0.024)	(0.024)
*R* ^2^	0.636	0.458	0.697	0.683
NT	23,480	23,135	11,082	11,081
*N*	7091	7015	4720	4719
HSC KWs versus non‐KWs	0.027	−0.025	0.003	0.013
	(0.017)	(0.017)	(0.022)	(0.023)
*R* ^2^	0.632	0.447	0.698	0.668
NT	33,187	32,673	15,838	15,834
*N*	10,143	10,041	6684	6682

*Note:* Sample includes waves 7–10 (pre‐pandemic) and wave 13. Outcome variables on financial stability are available from wave 7 onwards. Outcome variables on social interactions are available from wave 9. All model specifications incorporate IPWs to adjust for attrition. Controls included are age, education, marital status, number of children aged 0–15, household income, region dummies and interview year dummies. Estimates obtained using the unbalanced sample. Standard errors clustered at the household level. NT is the total number of observations and *N* is number of individual respondents in the sample.

Significance levels: ^+^ *p <* 0.10, **p < 0.05, ***p < 0.01.

## 6 Concluding Remarks

Using longitudinal data from the UKHLS, our analysis finds no statistically significant evidence that HSC KWs experienced worse mental health outcomes than other KWs or non‐ KWs during either the short‐ or medium‐term phases of the pandemic. This absence of differential effects should not be interpreted as evidence that HSC workers were unaffected and instead a sign of deteriorating mental health that was broadly shared across occupational groups. Importantly, HSC workers entered the pandemic already facing high levels of work‐related stress, burnout, and sickness absence (Brand et al. 2017; Toh et al. 2021). Long‐standing workforce pressures, underfunding, and systemic fragilities in the NHS and social care system meant that the health sector started the pandemic from a position of lower baseline well‐being (Brien and Keep 2023; Barrett 2024). The lack of a larger relative decline therefore likely reflects pre‐existing challenges within the sector, rather than an absence of strain during the pandemic.

Our results are robust to several specifications. We first examine whether differences exist in using different sample definitions based on waves or calendar year to uncover differential trends in mental health. We also restrict the sample to individuals who remained employed or who had no prior mental health issues, we find no robust differential effects across occupational groups, reinforcing the conclusion that the observed impacts are concentrated and limited in scope. Results are also robust to other definitions of mental health. Using the SF‐12 MCS we find no discernible effects across worker groups, with minor exceptions when looking at individual components of the score. However, item‐level differences observed in the GHQ and SF‐12 individual components are isolated and should be interpreted with caution. We also explored some potential mechanisms—namely, financial stability and so‐cial isolation—as possible channels through which the pandemic may have impacted mental health. While results using wave‐level data showed no significant effects, analyses based on annual data suggest small differences in perceived financial and social interactions between occupational groups.

Our results have several implications. First, the psychological and emotional risks faced by HSC workers should be understood in the context of structural vulnerabilities predating COVID‐19. Policies aimed at strengthening workforce well‐being in HSC must therefore look beyond the immediate pandemic response and address long‐standing organizational and resource constraints. Second, the lack of significant differences between HSC workers and other occupational groups highlights that mental health deterioration during the pandemic was widespread, reinforcing the need for universal mental health support rather than interventions narrowly targeted at a single sector. Third, high‐frequency mental health monitoring tools are essential for capturing rapid fluctuations in well‐being, especially during crises when aggregated annual data may mask short‐term dynamics. Finally, our findings emphasize the importance of long‐term workforce planning in HSC. Addressing chronic staffing shortages, reducing administrative pressures, and expanding access to mental health resources are crucial steps toward strengthening resilience in a sector that entered the pandemic already under severe strain.

Beyond the pandemic context, our findings have broader external validity for health, system, and labor market resilience. In this regard, we view the pandemic not only as a singular event but also as a blueprint for preparedness: policies that embed mental health capacity into crisis planning, maintain longitudinal monitoring of high‐risk groups, and preserve access to care during periods of constraints are likely to yield benefits under diverse future shocks. Our results depend on the context in which they have been estimated, but the underlying pathways are generalizable (Penninx et al. 2022).

Despite the strengths of our approach, several limitations should be acknowledged. First, while the UKHLS offers rich longitudinal data, the timing of interviews across and within waves introduces variability that may affect the identification of precise treatment periods — particularly in annual analyses where pre‐ and post‐pandemic responses may be aggregated. Second, our identification strategy relies on the assumption that all unobserved confounders are time‐invariant. Although we incorporate a rich set of observed covariates and apply inverse probability weights to mitigate attrition bias, the possibility of residual unobserved time‐varying confounding or selection effects cannot be entirely ruled out. This limitation is shared by most observational studies relying on parallel trends assumptions and should be kept in mind when interpreting our findings (Shahn and Hatfield 2024). Third, the classification of key workers is based on self‐reported sector information and may not fully capture individual‐ and role‐specific exposure to pandemic‐related stressors, especially within HSC. Lastly, our main mental health variables are self‐reported instruments, which, while validated, may not fully reflect clinical thresholds or account for variation in mental health literacy across groups.

## Funding

Jaime Pinilla acknowledges funding provided by the Plan Nacional de Investigación Científica y Técnica y de Innovación, Ministerio de Ciencia e Innovación, España (Grant Number: PID2021‐124067OB‐C22).

## Conflicts of Interest

The authors declare no conflicts of interest.

## Supporting information


Supporting Information S1

## Data Availability

The data that support the findings of this study are available upon request from the UK Data Service under Special License. The data are not publicly available due to privacy or ethical restrictions.

## Endnotes

^1^
SARS‐CoV‐2 infection itself is linked to post‐acute neuropsychiatric sequelae such as fatigue, cognitive impairment, and anxiety and depression that can extend months beyond infection (Penninx et al. 2022; Witteveen et al. 2023).

^2^
An early pandemic systematic review finds that among healthcare workers, 24%–65% reported anxiety, 12%–56% reported depression, and 30%–63% reported stress during the pandemic (Vizheh et al. 2020).

^3^
Wave 11 interviews were conducted between January 2019 and May 2021, while wave 12 interviews took place between January 2020 and May 2022.

^4^
Participation in the UKHLS COVID‐19 module was voluntary. As a result, certain groups tended to be over‐represented. To correct this, our estimates use the standard inverse‐probability weights in all specifications, as outlined in Section 4.5 below.

^5^
Figure A1 in the Supporting Information S1 shows the timeline of data collection of the main UKHLS and COVID‐19 surveys.

^6^
Figure 1c presents data for all COVID‐19 waves, including those waves (January, March and September 2021) excluded from the empirical analysis.

^7^
For example, the KW indicator variables classify NHS nurses working in hospitals (SOC code 2231 and SIC code 86101) and primary school teachers in state schools (with SOC 2314 and SIC code 85200) as KWs. The KW indicator variable will identify all other combinations of SOC/SIC corresponding to all essential workers (in health and social care, education and childcare, key public services, local and national government, food and other essential goods, public safety and national security, transport, utilities, communications, and financial services). Other non‐essential occupations such as chefs working in restaurants (SOC code 5434 and SIC 56101) will be coded as 0 in the KW variable. The indicator variable that identifies HSC workers and other KWs will take value 1 for professionals in this group (according to their SIC and SOC codes) and 0 for all other essential worker groups excluding HSC (education, transport, and all other non‐HSC groups). The indicator variable distinguishing HSC workers from non‐KWs is coded such that HSC workers are assigned a value of 1, while individuals employed in non‐essential occupations are assigned a value of 0.

^8^
KWs constitute approximately 33% of total workforce, with 31% of those classified as HSC KWs. See Tables 1a and 1b in ONS 2020.

^9^
The key‐worker sector question is only available in the May/June 2020 waves. In the July and September 2020 waves, it was asked only to respondents who had not participated in the previous wave. Given the short intervals between waves in the COVID‐19 survey, we impute missing values for worker classification using the closest available wave. For instance, July, September, and November 2020 values are inferred from the previous wave. This approach assumes that individuals did not change jobs within such short periods of time. This seems a reasonable assumption given the role they played during the pandemic.

^10^
Units are matched on the following pre‐treatment observable characteristics: age, gender, ethnicity, education, marital status, household size and income, employment status, physical health (as captured by SF‐12), regional variables and wave. The common support condition is satisfied. Observations in worker groups exhibit overlapping propensity score values, and each unit in the HSC group has a comparable control unit.

^11^
Prior to obtaining the regression estimates presented in Table A5 in the Supporting Information S1, we assess potential differences between respondents in the main survey and the COVID‐19 survey and replicate Figure 1c using the balanced sample. This is shown in Figure A4 in the Supporting Information S1. The mental health trends shown in the figure using the balanced sample closely mirror those in the original figure, suggesting comparability between samples.

^12^
Individuals that were present more than once for any given year were dropped. The post‐treatment variable starts after March 2020.

^13^
Using the SOC classification, we include as frontline workers the following worker groups: medical practitioners, radiographers, health professionals n.e.c, nurses, midwives, paramedics, health associate professionals n.e.c, nursing auxiliaries and assistants, ambulance staff (excluding paramedics), care workers and home carers, senior care workers.

^14^
For instance, we dichotomize the question *”Have you recently been able to concentrate on whatever you're doing?”* with value 1 if the respondent reports *”better than usual”* or *”same as usual”*. For questions such as *”Have you recently lost much sleep over worry?”*, we generate an indicator variable equal to 1 if responses are *”rather more than usual”* or *”much more than usual”*.

^15^
SF‐12 is not available in the COVID‐19 waves.

^16^
The specific questions ask “*During the past 4 weeks…: (1) How much of the time have you had any of the following problems with your work or other regular daily activities as a result of any emotional problems (such as feeling depressed or anxious)?* *(a) Accomplished less; (b) Did work or other activities less carefully than usual?; (2) Have you felt calm and peaceful?; (3) Have you felt downhearted and depressed?; (4) How much of the time has your physical health or emotional problems interfered with your social activities (like visiting friends, relatives)?*”. Responses to these questions are: all of the time, most of the time, some of the time, a little of the time, none of the time. We dichotomise these variables, assigning a value equal to 1 if the respondent answered all or most of the time, and 0 otherwise. These indicator variables reflect a worsened mental health outcome, except for the question on feeling calm for which a value of 1 reflects better mental health.

^17^
To create these indicator variables we exploit the following questions on financial stability available in the UKHLS from wave 7: *”How well would you say you yourself are managing financially these days?”*, with possible answers being: living comfortably, doing alright, just about getting by, finding it quite difficult, finding it very difficult.; *”Looking ahead, how do you think you will be financially a year from now, will you be…”*, with the following answers: better off, worse of than now, about the same. The information on social engagement is based on these two questions available only from wave 9: *”How often do you feel lonely?”* and *”How often do you feel isolated from others?”*, with possible answers: hardly ever or never, some of the time, or often. For the purpose of our analysis we generated dummies the following dummies: (1) Current finance: taking value 1 if living comfortably/doing alright and 0 otherwise; (2) Future finance coded as 1 if answered same or better and 0 if worse; (3) Lonely, taking value 1 if hardly ever/never and 0 otherwise; (4) Isolated coded as 1 if hardly ever/never and 0 otherwise.

^18^
The variable on current financial stability is available in waves April, May, July and November 2020. The variable on future financial stability is available for April, May and July 2020. The variable on isolation is available in the 2020 waves. The variable on future financial stability was rephrased to *”Looking ahead, how do you think you will be financially a month from now, will you be…”*, changing the time reference from yearly (as in the main questionnaire) to monthly. The question on loneliness was modified to *”In the last 4 weeks, how often did you feel lonely?”*.
