# Supplementary material for: The Mental Health Impact of the COVID‐19 Pandemic on Health and Social Care Workers
Source: Health Econ. 2026 Mar 9;35(6):960–77. doi: 10.1002/hec.70090 (PMC13126099; doi:10.1002/hec.70090)
Supplement: Supplementary file 1 — Supporting Information S1 [file HEC-35-960-s001.docx]

# Appendix

## Tables

### Table A1: GHQ individual questions

Item Label Question Options

|  | Have you recently... |  | 1 | 2 | 3 | 4 |  |
| --- | --- | --- | --- | --- | --- | --- | --- |
| 1 Concentration | been able to concentrate on whatever you’re doing? |  | Better than usual | Same as usual | Less than usual | Much less than usual |  |
| 2 Loss of sleep | lost much sleep over worry? |  | Not at all | No more than usual | Rather more than usual | Much more than usual |  |
| 3 Playing a useful role | felt that you were playing a useful part in things? |  | More than usual | Same as usual | Less so than usual | Much less than usual |  |
| 4 Capable of making decisions | felt capable of making decisions? |  | More so than usual | Same as usual | Less so than usual | Much less capable |  |
| 5 Constantly under strain | felt constantly under strain? |  | Not at all | No more than usual | Rather more than usual | Much more than usual |  |
| 6 Problem overcoming difficulties | felt you couldn’t overcome your difficulties? |  | Not at all | No more than usual | Rather more than usual | Much more than usual |  |
| 7 Enjoy day-to-day activities | been able to enjoy your normal day-to-day activities? |  | More than usual | Same as usual | Less so than usual | Much less than usual |  |
| 8 Ability to face problems | been able to face up to problems? |  | More so than usual | Same as usual | Less so than usual | Much less able |  |
| 9 Unhappy or depressed | been feeling unhappy or depressed? |  | Not at all | No more than usual | Rather more than usual | Much more than usual |  |
| 10 Losing confidence | been losing confidence in yourself? |  | Not at all | No more than usual | Rather more than usual | Much more than usual |  |
| 11 Believe in self-worth | been thinking of yourself as a worthless person? |  | Not at all | No more than usual | Rather more than usual | Much more than usual |  |
| 12 General Happiness | been feeling happy, all things considered? |  | More so than usual | About the same as usual | Less so than usual | Much less than usual |  |

### Table A2: Descriptive Statistics - Main dataset

| Pre-2020 | | | Post-2020 | | | |
| --- | --- | --- | --- | --- | --- | --- |
|  | Mean | Std.Dev |  | Mean | Std.Dev |  |
| KWs | 0.466 | 0.499 |  | 0.464 | 0.499 |  |
| HSC KWs | 0.237 | 0.425 |  | 0.248 | 0.432 |  |
| GHQ Likert | 10.829 | 5.182 |  | 11.594 | 5.391 |  |
| GHQ Caseness | 1.612 | 2.845 |  | 1.866 | 3.110 |  |
| Distressed | 0.146 | 0.353 |  | 0.173 | 0.379 |  |
| Severely Dis | 0.061 | 0.239 |  | 0.078 | 0.269 |  |
| Age | 40.875 | 13.298 |  | 44.573 | 12.780 |  |
| Male | 0.449 | 0.497 |  | 0.430 | 0.495 |  |
| White | 0.828 | 0.377 |  | 0.865 | 0.342 |  |
| Mixed | 0.023 | 0.149 |  | 0.022 | 0.147 |  |
| Asian/Asian British | 0.096 | 0.295 |  | 0.074 | 0.261 |  |
| Black/Black British | 0.043 | 0.204 |  | 0.032 | 0.177 |  |
| Other | 0.010 | 0.098 |  | 0.007 | 0.083 |  |
| Degree or higher | 0.510 | 0.500 |  | 0.573 | 0.495 |  |
| Secondary | 0.239 | 0.427 |  | 0.215 | 0.411 |  |
| Other | 0.228 | 0.420 |  | 0.197 | 0.398 |  |
| No qualification | 0.022 | 0.147 |  | 0.015 | 0.120 |  |
| Married/partner | 0.516 | 0.500 |  | 0.568 | 0.495 |  |
| Single | 0.375 | 0.484 |  | 0.317 | 0.465 |  |
| Separated/divorced | 0.097 | 0.296 |  | 0.101 | 0.302 |  |
| Widowed | 0.012 | 0.107 |  | 0.013 | 0.115 |  |
| Children aged 0-15 | 0.704 | 0.980 |  | 0.625 | 0.918 |  |
| Net Household income | 8.102 | 0.576 |  | 8.214 | 0.579 |  |
| North East | 0.033 | 0.179 |  | 0.035 | 0.184 |  |
| North West | 0.105 | 0.306 |  | 0.105 | 0.307 |  |
| Yorkshire | 0.083 | 0.275 |  | 0.084 | 0.278 |  |
| East Midlands | 0.077 | 0.267 |  | 0.073 | 0.259 |  |
| West Midlands | 0.086 | 0.280 |  | 0.081 | 0.273 |  |
| East of England | 0.086 | 0.281 |  | 0.092 | 0.289 |  |
| London | 0.122 | 0.328 |  | 0.100 | 0.300 |  |
| South East | 0.127 | 0.333 |  | 0.129 | 0.335 |  |
| South West | 0.086 | 0.280 |  | 0.094 | 0.291 |  |
| Wales | 0.061 | 0.239 |  | 0.065 | 0.246 |  |
| Scotland | 0.082 | 0.274 |  | 0.085 | 0.279 |  |
| Northern Ireland | 0.053 | 0.225 |  | 0.057 | 0.232 |  |
| N | 45701 |  |  | 7303 |  |  |

*Note:* This table shows the descriptive statistics of variables pre-pandemic (Waves 7 to 10) and post-pandemic (Wave 13). GHQ Likert ranges from 0 (the least distressed) to 36 (the most distressed). GHQ Caseness ranges from

0 (the least distressed) to 12 (the most distressed). Net household income expressed in logarithmic terms.

### Table A3: Descriptive Statistics - COVID-19 dataset

| Pre-2020 | | | Post-2020 | | | |
| --- | --- | --- | --- | --- | --- | --- |
|  | Mean | Std.Dev |  | Mean | Std.Dev |  |
| KWs | 0.467 | 0.499 |  | 0.473 | 0.499 |  |
| HSC KWs | 0.237 | 0.425 |  | 0.321 | 0.467 |  |
| GHQ Likert | 10.834 | 5.186 |  | 12.214 | 5.689 |  |
| GHQ Caseness | 1.613 | 2.846 |  | 2.388 | 3.275 |  |
| Distressed | 0.146 | 0.353 |  | 0.217 | 0.412 |  |
| Severely Distressed | 0.061 | 0.239 |  | 0.095 | 0.294 |  |
| Age | 40.927 | 13.284 |  | 47.166 | 12.390 |  |
| Male | 0.449 | 0.497 |  | 0.415 | 0.493 |  |
| White | 0.828 | 0.378 |  | 0.906 | 0.291 |  |
| Mixed | 0.023 | 0.149 |  | 0.018 | 0.134 |  |
| Asian/Asian British | 0.096 | 0.295 |  | 0.051 | 0.221 |  |
| Black/Black British | 0.043 | 0.204 |  | 0.020 | 0.140 |  |
| Other | 0.010 | 0.099 |  | 0.004 | 0.065 |  |
| Degree or higher | 0.511 | 0.500 |  | 0.583 | 0.493 |  |
| Secondary | 0.238 | 0.426 |  | 0.207 | 0.405 |  |
| Other | 0.229 | 0.420 |  | 0.198 | 0.398 |  |
| No qualification | 0.022 | 0.147 |  | 0.012 | 0.110 |  |
| Married/partner | 0.517 | 0.500 |  | 0.595 | 0.491 |  |
| Single | 0.374 | 0.484 |  | 0.277 | 0.447 |  |
| Separated/divorced | 0.097 | 0.296 |  | 0.116 | 0.321 |  |
| Widowed | 0.012 | 0.107 |  | 0.012 | 0.110 |  |
| Children aged 0-15 | 0.704 | 0.981 |  | 0.615 | 0.916 |  |
| Net Household income | 8.100 | 0.576 |  | 8.089 | 0.995 |  |
| North East | 0.033 | 0.179 |  | 0.034 | 0.182 |  |
| North West | 0.105 | 0.306 |  | 0.099 | 0.299 |  |
| Yorkshire | 0.083 | 0.275 |  | 0.082 | 0.275 |  |
| East Midlands | 0.077 | 0.267 |  | 0.076 | 0.265 |  |
| West Midlands | 0.086 | 0.280 |  | 0.083 | 0.276 |  |
| East of England | 0.087 | 0.281 |  | 0.099 | 0.299 |  |
| London | 0.122 | 0.328 |  | 0.093 | 0.290 |  |
| South East | 0.127 | 0.333 |  | 0.142 | 0.349 |  |
| South West | 0.086 | 0.280 |  | 0.102 | 0.303 |  |
| Wales | 0.060 | 0.238 |  | 0.055 | 0.228 |  |
| Scotland | 0.081 | 0.273 |  | 0.092 | 0.289 |  |
| Northern Ireland | 0.053 | 0.224 |  | 0.043 | 0.203 |  |
| N | 45719 |  |  | 33110 |  |  |

*Note:* This table shows the descriptive statistics pre-pandemic (Waves 7 to 10) and post-pandemic (April, May, June, July, September and November 2020). The sample size pre-2020 differs to that in Table A2 due to different obser- vations being dropped in the fixed effect model. Note that a smaller number of respondents from the main UKHLS survey responded in COVID-19 waves. GHQ Likert ranges from 0 (the least distressed) to 36 (the most distressed). GHQ Caseness ranges from 0 (the least distressed) to 12 (the most distressed). Net household income expressed in logarithmic terms.

### Table A4: The impact of the pandemic on mental health

| (1) | | (2) | (3) | (4) |
| --- | --- | --- | --- | --- |
| GHQ Likert | | GHQ Caseness | Distress | Severe Distress |
| Panel A: COVID-19 Waves | |  |  |  |
| COVID-19 | 1.070*** | 0.745*** | 0.068*** | 0.025 |
|  | (0.269) | (0.159) | (0.021) | (0.016) |
| *R*2 | 0.601 | 0.549 | 0.481 | 0.443 |
| NT | 78829 | 78829 | 78829 | 78829 |
| N | 17370 | 17370 | 17370 | 17370 |
| Panel B: Main Waves | | | | |
| COVID-19 | 0.661*** | 0.455*** | 0.036*** | 0.014** |
|  | (0.100) | (0.060) | (0.008) | (0.006) |
| *R*2 | 0.572 | 0.516 | 0.459 | 0.410 |
| NT | 75538 | 75538 | 75538 | 75538 |
| N | 17341 | 17341 | 17341 | 17341 |

*Note:* Sample in Panel A includes waves 7 to 10 (pre-pandemic) and waves April to November 2020 (post-pandemic). Sample in Panel B includes waves 7 to 10 (pre-pandemic) and wave 13 (post-pandemic). Estimates obtained using the same sample employed in the DID analysis. Estimates are the result of regressing mental health outcomes on a dummy post-pandemic, controlling for age, education, marital status, number of children aged 0 to 15, household income, region dummies and interview year dummies. All model specifications incorporate IPWs to adjust for attrition. Standard errors clustered at the household level. NT is the total number of observations and N is number of individual respondents in the sample. Panel A sample size is larger than Panel B due to Panel A employing six waves and Panel B relying on data from a single wave 13. Significance levels: +*p <* 0*.*10, ∗∗ *p <* 0*.*05, ∗∗∗ *p <* 0*.*01.

### Table A5: Effects of the Pandemic on Mental Health - Short-term Effects

|  | (1)  GHQ Likert | (2)  GHQ Caseness | (3)  Distress | (4)  Severe Distress |
| --- | --- | --- | --- | --- |
| HSC KWs vs other KWs | 0.487** | 0.220 | 0.036** | 0.008 |
|  | (0.234) | (0.137) | (0.018) | (0.012) |

*R*^2^ 0.609 0.549 0.477 0.434

| NT | 14721 | 14721 | 14721 | 14721 |
| --- | --- | --- | --- | --- |
| N | 2707 | 2707 | 2707 | 2707 |
| HSC KWs vs Non-KWs | -0.091 | -0.177 | -0.008 | -0.014 |
|  | (0.213) | (0.126) | (0.017) | (0.011) |

*R*^2^ 0.603 0.546 0.464 0.455

| NT | 20217 | 20217 | 20217 | 20217 |
| --- | --- | --- | --- | --- |
| N | 3497 | 3497 | 3497 | 3497 |

*Note:* Sample includes waves 7 to 10 (pre-pandemic) and waves April to November 2020 (post-pandemic). All model specifications incorporate IPWs to adjust for attrition. Controls included are age, education, marital status, number of children aged 0 to 15, household income, region dummies and interview year dummies. Estimates obtained using the balanced sample. Standard errors clustered at the household level. NT is the total number of observations and N is number of individual respondents in the sample.

Significance levels: +*p <* 0*.*10, ∗∗ *p <* 0*.*05, ∗∗∗ *p <* 0*.*01.

### Table A6: Effects of the Pandemic on Mental Health - Medium-term Effects

|  | (1)  GHQ Likert | (2)  GHQ Caseness | (3)  Distress | (4)  Severe Distress |
| --- | --- | --- | --- | --- |
| HSC KWs vs other KWs | 0.083 | 0.031 | -0.010 | 0.002 |
|  | (0.278) | (0.168) | (0.021) | (0.016) |

*R*^2^ 0.602 0.550 0.491 0.451

| NT | 13323 | 13323 | 13323 | 13323 |
| --- | --- | --- | --- | --- |
| N | 3637 | 3637 | 3637 | 3637 |
| HSC KWs vs non-KWs | 0.174 | 0.150 | 0.017 | 0.021 |
|  | (0.263) | (0.161) | (0.020) | (0.015) |

*R*^2^ 0.609 0.552 0.489 0.458

| NT | 17914 | 17914 | 17914 | 17914 |
| --- | --- | --- | --- | --- |
| N | 4837 | 4837 | 4837 | 4837 |

*Note:* Sample includes waves 7 to 10 (pre-pandemic) and wave 13 (post-pandemic). All model specifications incorporate IPWs to adjust for attrition. Controls included are age, education, marital status, number of children aged 0 to 15, household income, region dummies and interview year dummies. Esti- mates obtained using the balanced sample. Standard errors clustered at the household level. NT is the total number of observations and N is number of individual respondents in the sample. Significance levels:

+*p <* 0*.*10, ∗∗ *p <* 0*.*05, ∗∗∗ *p <* 0*.*01.

### Table A7: Effects of the Pandemic on Mental Health - LDV Approach

|  | (1)  GHQ Likert | (2)  GHQ Caseness | (3)  Distress | (4)  Severe Distress |
| --- | --- | --- | --- | --- |
| HSC KWs vs other KWs | 0.032 | 0.037 | 0.000 | -0.008 |
|  | (0.165) | (0.099) | (0.013) | (0.009) |

*R*^2^ 0.220 0.164 0.114 0.075

| *N* | 4756 | 4756 | 4756 | 4756 |
| --- | --- | --- | --- | --- |
| HSC KWs vs non-KWs | 0.113 | 0.057 | 0.008 | 0.004 |
|  | (0.157) | (0.095) | (0.012) | (0.009) |

*R*^2^ 0.234 0.175 0.120 0.069

*N* 6781 6781 6781 6781

*Note:* Estimates presented are based on a regression of mental health outcomes in wave 13 on the mental health outcomes in waves 7, 8, 9 and 10. Observations included in this model are therefore limited to those respondents present in waves 7-10 and 13. All specifications are adjusted by the IPW and, for consistency with the set of control variables in Table 2, we include age, education, marital status, number of children aged 0 to 15, household income, region and interview year dummies. Standard errors clustered at the household level. NT is the total number of observations and N is number of individual respondents in the

sample. Significance levels: +*p <* 0*.*10, ∗∗ *p <* 0*.*05, ∗∗∗ *p <* 0*.*01.

### Table A8: Effects of the Pandemic on Mental Health - Matching based on pre-treatment variables

|  | (1)  GHQ Likert | (2)  GHQ Caseness | (3)  Distress | (4)  Severely Distressed |
| --- | --- | --- | --- | --- |
| HSC KWs vs other KWs | -0.274 | -0.199 | -0.027 | -0.016 |
|  | (0.248) | (0.154) | (0.019) | (0.015) |
| *R*2 | 0.614 | 0.564 | 0.513 | 0.469 |
| NT | 15997 | 15997 | 15997 | 15997 |
| N | 4705 | 4705 | 4705 | 4705 |
| HSC KWs vs non-KWs | 0.209 | 0.141 | 0.025 | 0.021 |
|  | (0.241) | (0.148) | (0.019) | (0.014) |
| *R*2 | 0.620 | 0.562 | 0.512 | 0.461 |
| NT | 17385 | 17385 | 17385 | 17385 |
| N | 5085 | 5085 | 5085 | 5085 |

*Note:* Sample includes observations matched on pre-treatment observable characteristics: age, gender, ethnicity, education, marital status, household size and income, employment status, mental and physical health (as captured by SF-12), regional variables and wave. All DID specifications include IPWs and, for consistency with the set of control variables in Table 2, control for age, education, marital status, number of children aged 0 to 15, household income, region and interview year dummies. Standard errors clustered at the household level. NT is the total number of observations and N is number of individual respondents in the sample. Significance levels: +*p <* 0*.*10, ∗∗ *p <* 0*.*05, ∗∗∗ *p <* 0*.*01.

### Table A9: Respondents with no severe psychological distress pre-pandemic

|  | (1)  GHQ Likert | (2)  GHQ Caseness | (3)  Distress | (4)  Severe Distress |
| --- | --- | --- | --- | --- |
| HSC KWs vs other KWs | -0.091 | -0.061 | -0.011 | 0.000 |
|  | (0.213) | (0.130) | (0.017) | (0.010) |
| *R*2 | 0.580 | 0.474 | 0.420 | 0.317 |
| NT | 19382 | 19382 | 19382 | 19382 |
| N | 5859 | 5859 | 5859 | 5859 |
| HSC KWs vs non-KWs | -0.064 | 0.043 | 0.007 | 0.009 |
|  | (0.200) | (0.121) | (0.016) | (0.010) |
| *R*2 | 0.581 | 0.480 | 0.421 | 0.324 |
| NT | 27150 | 27150 | 27150 | 27150 |
| N | 8341 | 8341 | 8341 | 8341 |

*Note:* Sample includes waves 7 to 10 (pre-pandemic) and wave 13 (post-pandemic). Sample restricted to those individuals with no severe distress (GHQ *>* 20) in waves 7 to 10 prior to the pandemic. All model specifications incorporate IPWs to adjust for attrition. Controls included are age, education, marital status, number of children aged 0 to 15, household income, region dummies and interview year dummies. Estimates obtained using the unbalanced sample. Standard errors clustered at the household level. NT is the total number of observations and N is number of individual respondents in the sample. Significance

levels: +*p <* 0*.*10, ∗∗ *p <* 0*.*05, ∗∗∗ *p <* 0*.*01.

### Table A10: Effects of the Pandemic on Mental Health - Heterogenous Effects by Gender

Females Males

|  | (1) | (2) | (3) | (4) |  | (5) | (6) | (7) | (8) |  |
| --- | --- | --- | --- | --- | --- | --- | --- | --- | --- | --- |
|  | GHQ Likert | GHQ Caseness | Distress | Severe Distress |  | GHQ Likert | GHQ Caseness | Distress | Severe Distress |  |
| HSC KWs vs other KWs | -0.269 | -0.151 | -0.019 | -0.014 |  | 0.352 | -0.009 | -0.036 | 0.023 |  |
|  | (0.266) | (0.166) | (0.021) | (0.016) |  | (0.477) | (0.268) | (0.036) | (0.023) |  |
| *R*2 | 0.616 | 0.571 | 0.519 | 0.480 |  | 0.616 | 0.561 | 0.521 | 0.444 |  |
| NT | 14100 | 14100 | 14100 | 14100 |  | 8942 | 8942 | 8942 | 8942 |  |
| N | 4263 | 4263 | 4263 | 4263 |  | 2736 | 2736 | 2736 | 2736 |  |
| HSC KWs vs non-KWs | 0.249 | 0.192 | 0.038+ | 0.022 |  | -0.139 | -0.254 | -0.053 | -0.004 |  |
|  | (0.251) | (0.156) | (0.020) | (0.015) |  | (0.460) | (0.254) | (0.034) | (0.022) |  |
| *R*2 | 0.626 | 0.569 | 0.519 | 0.471 |  | 0.606 | 0.562 | 0.513 | 0.470 |  |
| NT | 18286 | 18286 | 18286 | 18286 |  | 14265 | 14265 | 14265 | 14265 |  |
| N | 5621 | 5621 | 5621 | 5621 |  | 4392 | 4392 | 4392 | 4392 |  |

*Note:* Sample includes waves 7 to 10 (pre-pandemic) and wave 13 (post-pandemic). Sub-group analysis by gender: Columns (1) to (4) use the sample for females; Columns (5) to (8) use the sample for males only. All model specifications incorporate IPWs to adjust for attrition. Controls included are age, education, marital status, number of children aged 0 to 15, household income, region dummies and interview year dummies. Estimates obtained using the unbalanced sample. Standard errors clustered at the household level. NT is the total number of observations and N is number of individual respondents in the sample. Significance levels: + *p <* 0*.*10, ∗∗ *p <* 0*.*05, ∗∗∗ *p <* 0*.*01.

### Table A11: Effects of the Pandemic on Mental Health - Heterogenous Effects by Ethnic Group

Ethnic Minority White

|  | (1) | (2) | (3) | (4) |  | (5) | (6) | (7) | (8) |  |
| --- | --- | --- | --- | --- | --- | --- | --- | --- | --- | --- |
|  | GHQ Likert | GHQ Caseness | Distress | Severe Distress |  | GHQ Likert | GHQ Caseness | Distress | Severe Distress |  |
| HSC KWs vs other KWs | 0.867 | 0.319 | 0.033 | 0.058+ |  | -0.254 | -0.136 | -0.023 | -0.013 |  |
|  | (0.538) | (0.326) | (0.042) | (0.032) |  | (0.247) | (0.152) | (0.019) | (0.014) |  |
| *R*2 | 0.630 | 0.580 | 0.543 | 0.480 |  | 0.618 | 0.570 | 0.518 | 0.469 |  |
| NT | 4150 | 4150 | 4150 | 4150 |  | 18893 | 18893 | 18893 | 18893 |  |
| N | 1351 | 1351 | 1351 | 1351 |  | 5648 | 5648 | 5648 | 5648 |  |
| HSC KWs vs non-KWs | 0.984+ | 0.410 | 0.057 | 0.079*** |  | -0.002 | 0.081 | 0.009 | 0.009 |  |
|  | (0.522) | (0.310) | (0.039) | (0.030) |  | (0.235) | (0.144) | (0.018) | (0.014) |  |
| *R*2 | 0.655 | 0.613 | 0.568 | 0.516 |  | 0.614 | 0.561 | 0.509 | 0.463 |  |
| NT | 5371 | 5371 | 5371 | 5371 |  | 27177 | 27177 | 27177 | 27177 |  |
| N | 1788 | 1788 | 1788 | 1788 |  | 8224 | 8224 | 8224 | 8224 |  |

*Note:* Sample includes waves 7 to 10 (pre-pandemic) and wave 13 (post-pandemic). Sub-group analysis by ethnic group: Columns (1) to (4) use the sample for respondents of ethnic minority background; Columns (5) to (8) use the sample for white respondents. All model specifications incorporate IPWs to adjust for attrition. Controls included are age, education, marital status, number of children aged 0 to 15, household income, region dummies and interview year dummies. Estimates obtained using the unbalanced sample. Standard errors clustered at the household level. NT is the total number of observations and N is number of individual respondents in the sample. Significance levels: + *p <* 0*.*10,

∗∗ *p <* 0*.*05, ∗∗∗ *p <* 0*.*01.

34

### Table A12: Effects of the Pandemic on Mental Health - GHQ Individual Questions

| (1) | (2) | (3) | (4) | (5) | (6) | (7) | (8) | (9) | (10) | (11) | (12) |
| --- | --- | --- | --- | --- | --- | --- | --- | --- | --- | --- | --- |
| Concentration | Sleep | Role | Decisions | Strain | Difficulties | Enjoy activities | Face Probs | Unhappy | Confidence | Worthless | Happiness |
| HSC KWs vs other KWs -0.006 | -0.012 | 0.016 | 0.001 | 0.021 | -0.002 | -0.010 | -0.010 | -0.022 | -0.025 | -0.026+ | 0.000 |
| (0.019) | (0.019) | (0.016) | (0.014) | (0.021) | (0.017) | (0.019) | (0.015) | (0.020) | (0.018) | (0.013) | (0.017) |
| *R*^2^ 0.466 | 0.467 | 0.468 | 0.460 | 0.493 | 0.463 | 0.470 | 0.469 | 0.476 | 0.500 | 0.501 | 0.467 |
| NT 23116 | 23121 | 23106 | 23119 | 23122 | 23116 | 23121 | 23117 | 23113 | 23112 | 23109 | 23115 |
| N 7015 | 7014 | 7013 | 7015 | 7017 | 7014 | 7016 | 7015 | 7013 | 7012 | 7010 | 7015 |
| HSC KWs vs non-KWs -0.012 | -0.001 | -0.007 | -0.011 | 0.043** | 0.018 | -0.015 | -0.020 | 0.005 | 0.009 | -0.007 | -0.010 |
| (0.018) | (0.018) | (0.015) | (0.013) | (0.020) | (0.016) | (0.018) | (0.014) | (0.018) | (0.017) | (0.012) | (0.016) |
| *R*^2^ 0.469 | 0.458 | 0.452 | 0.454 | 0.496 | 0.470 | 0.462 | 0.463 | 0.476 | 0.508 | 0.496 | 0.467 |
| NT 32655 | 32672 | 32652 | 32670 | 32672 | 32658 | 32666 | 32665 | 32663 | 32662 | 32667 | 32656 |
| N 10034 | 10040 | 10035 | 10039 | 10039 | 10035 | 10038 | 10036 | 10036 | 10035 | 10036 | 10033 |

*Notes:* Sample includes waves 7 to 10 (pre-pandemic) and wave 13 (post-pandemic). All model specifications incorporate IPWs to adjust for attrition. Controls included are age, education, marital status, number of children aged 0 to 15, household income, region dummies and interview year dummies. Estimates obtained using the unbalanced sample. Standard errors clustered at the household level. NT is the total number of observations and N is number of individual respondents in the sample. Significance levels: + *p <* 0*.*10, ∗∗ *p <* 0*.*05, ∗∗∗ *p <* 0*.*01.

### Table A13: Mechanisms - Yearly data

|  | (1)  Current Finances | (2)  Future Finances | (3)  Loneliness | (4)  Isolation |
| --- | --- | --- | --- | --- |
| HSC KW vs other KW | 0.025** | 0.012 | 0.022 | 0.025 |
|  | (0.011) | (0.010) | (0.015) | (0.016) |
| *R*2 | 0.606 | 0.413 | 0.643 | 0.618 |
| NT | 32924 | 32506 | 19102 | 19106 |
| N | 8182 | 8110 | 6042 | 6044 |
| HSC KW vs non-KW | 0.032*** | 0.010 | 0.035** | 0.031** |
|  | (0.011) | (0.010) | (0.014) | (0.015) |
| *R*2 | 0.602 | 0.408 | 0.647 | 0.611 |
| NT | 46553 | 45920 | 27243 | 27249 |
| N | 11752 | 11652 | 8665 | 8668 |

*Note:* Sample constructed based on Waves 7 to 13 (including waves 11 and 12) and grouped by calendar year of interviewed, from 2015 to 2022. All model specifications incorporate IPWs to adjust for attrition. Controls included are age, education, marital status, number of children aged 0 to 15, household income, region dummies and interview year dummies. Estimates obtained using the unbalanced sample. Standard

errors clustered at the household level. NT is the total number of observations and N is number of individual respondents in the sample. Significance levels: +*p <* 0*.*10, ∗∗ *p <* 0*.*05, ∗∗∗ *p <* 0*.*01.

## Figures

### Figure A1: UKHLS Timeline of Data Collection


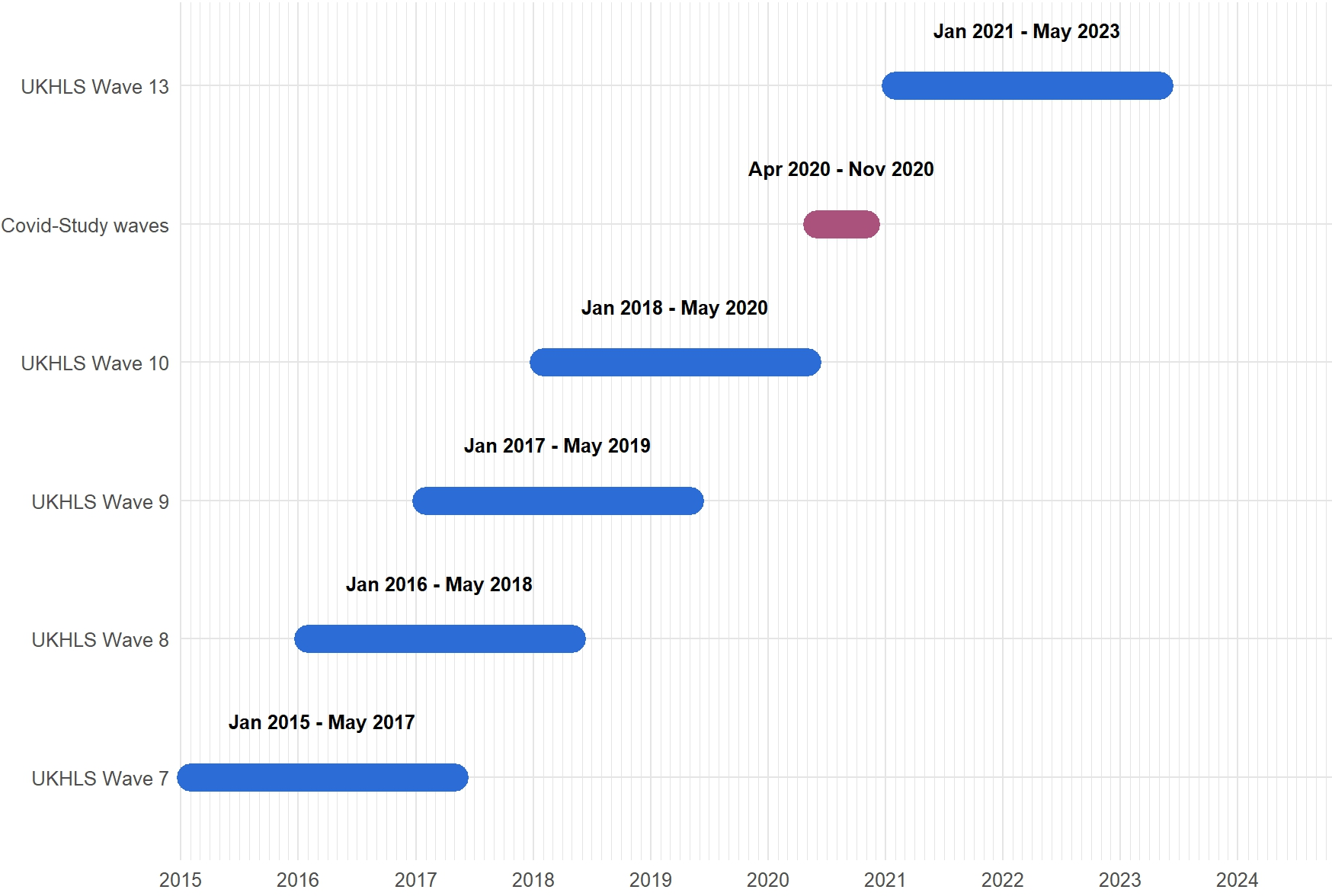


*Notes*: This figure is an adaptation of the timeline for data collection available in the UKHLS website (University of Essex & Research 2024), including the survey collection of the main survey with the timeline for data collection carried out for the COVID-19 surveys. Waves included in the Figure are those employed in the analysis.

### Figure A2: Trends in Mental Health for HSC KWs vs Other KWs

(a) GHQ-Likert (b) GHQ-Caseness


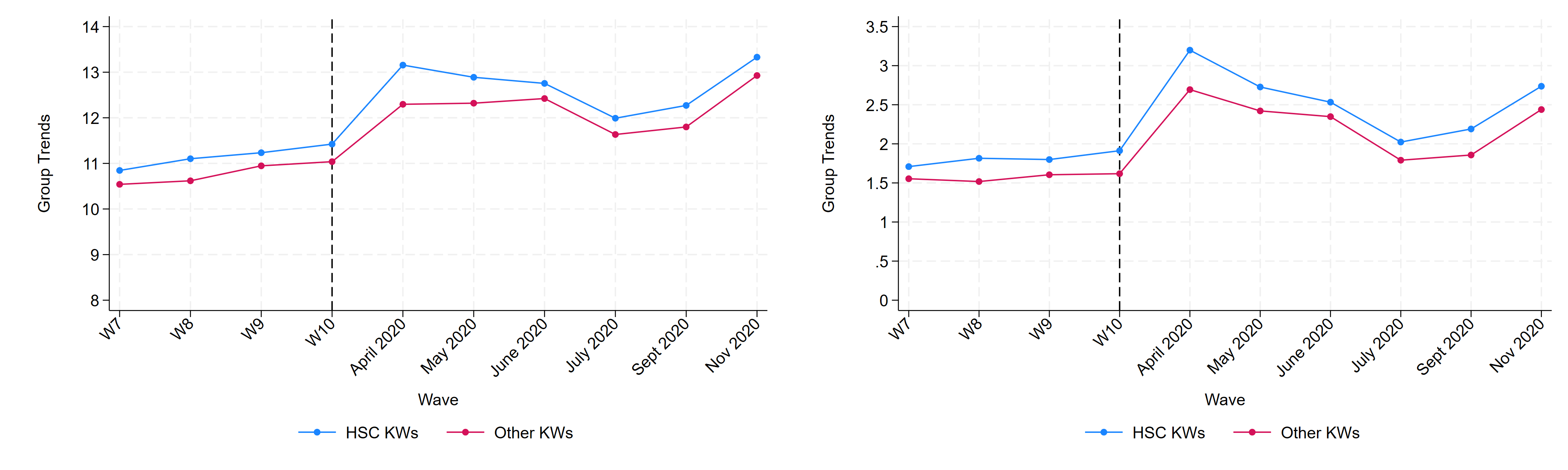


(c) Distress (d) Severely Distressed


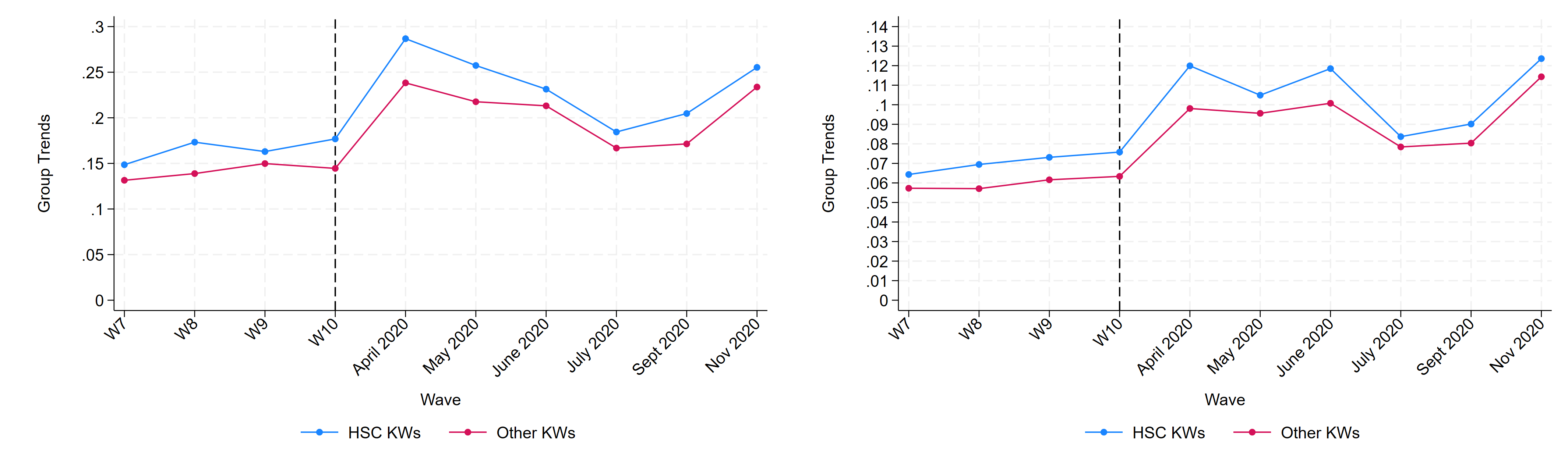


*Notes*: Figures show trends for all measures of mental health examined in the empirical analysis for the treatment group HSC KWs and control group Other KWs. Sample employed to generate the figures comprises KWs participating in waves 7 to 10 (pre-pandemic) and waves April to November 2020 (post-pandemic). The vertical dotted line indicates the wave prior to the onset of the pandemic.

### Figure A3: Trends in Mental Health for HSC KWs vs Non-KWs

(a) GHQ-Likert (b) GHQ-Caseness


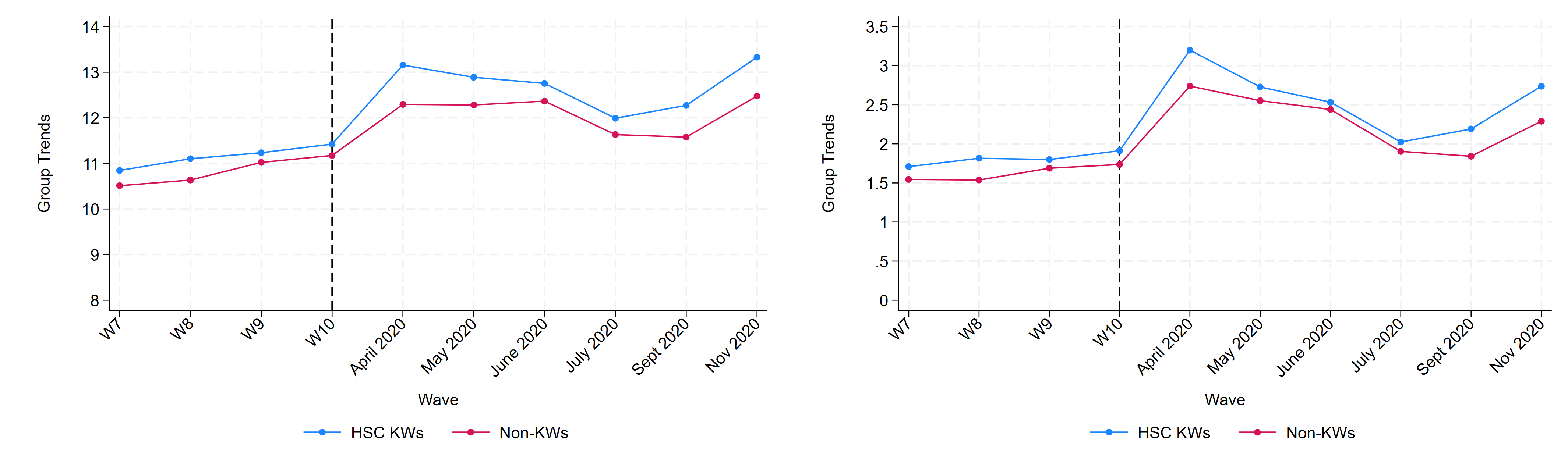


(c) Distress (d) Severely Distressed


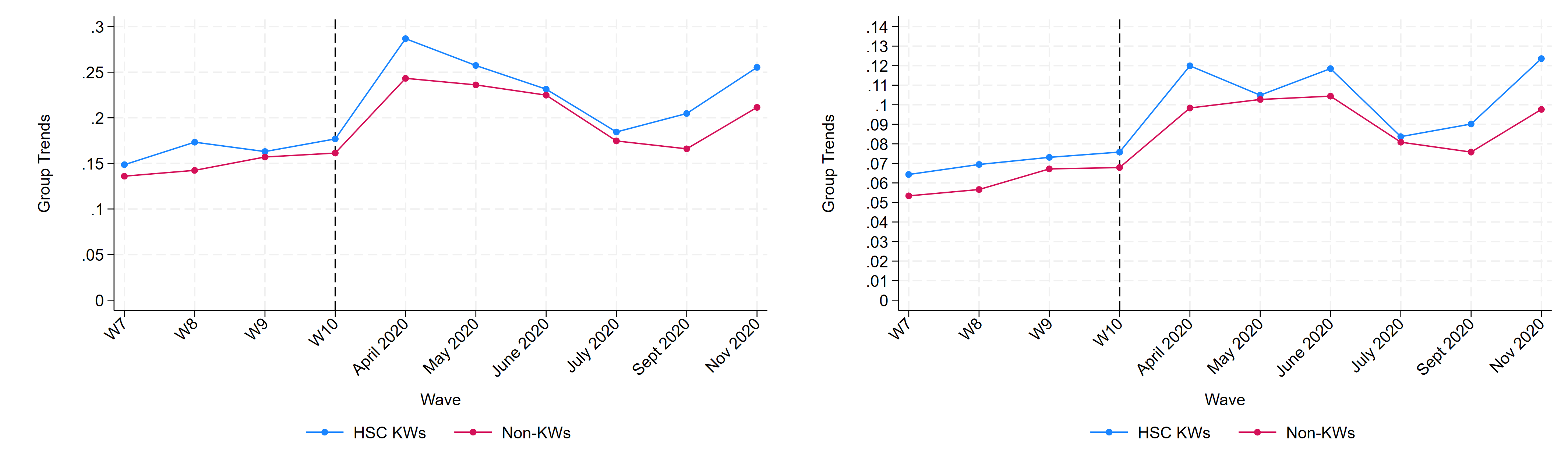


*Notes*: Figures show trends for all measures of mental health examined in the empirical analysis for the treatment group HSC KWs and control group non-KWs. Sample employed to generate the figures comprises KWs participating in waves 7 to 10 (pre-pandemic) and waves April to November 2020 (post-pandemic). The vertical dotted line indicates the wave prior to the onset of the pandemic.

### Figure A4: Trends in mental health - COVID-19 balanced sample


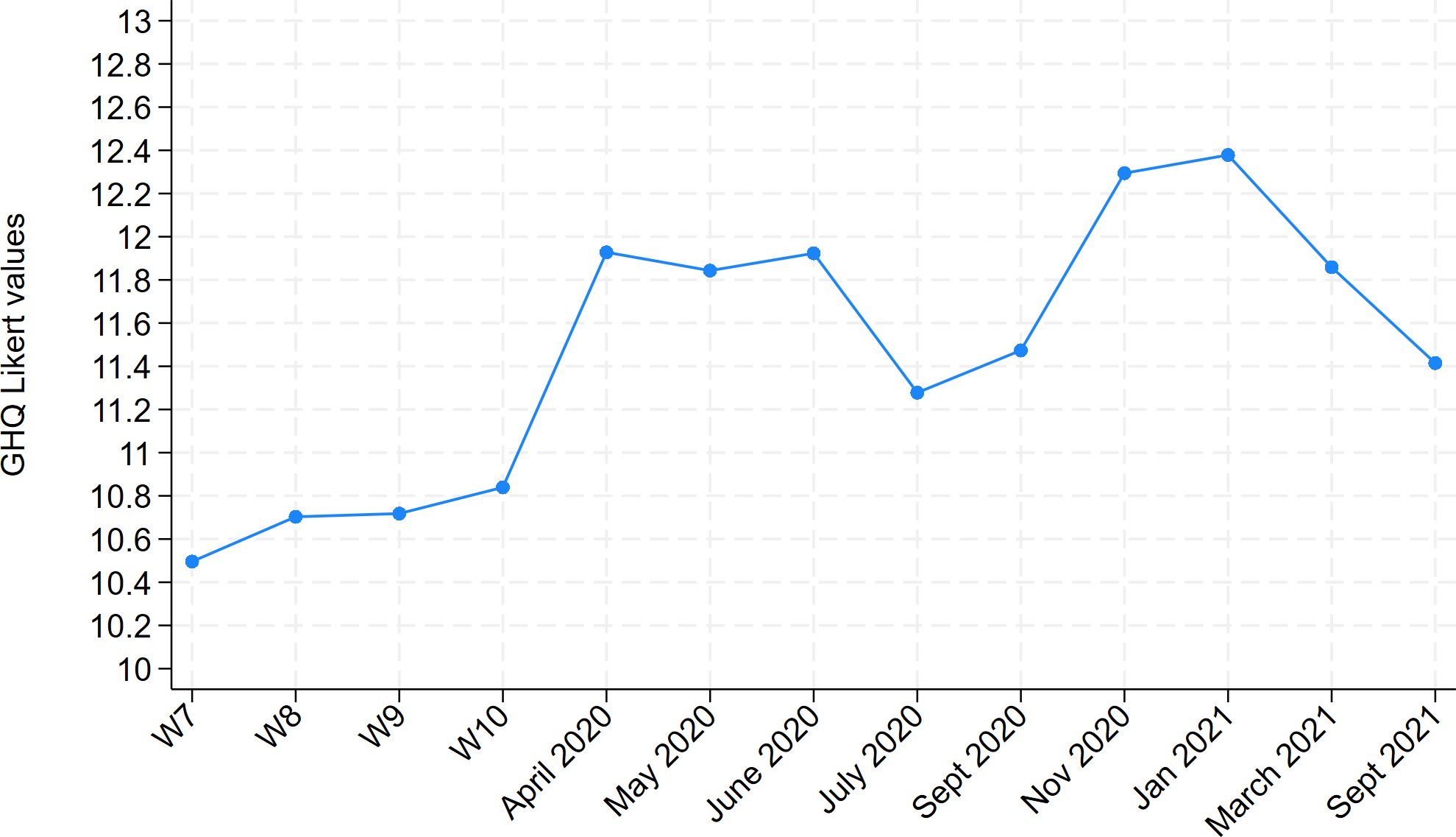


*Notes*: This figure shows trends in mental health (measured by the GHQ Likert variable). The graph is based on the balanced sample, constructed by pooling Waves 7–10 (pre-pandemic) with all COVID-19 waves (post-pandemic).

### Figure A5: Heterogeneous effects HSC KWs vs Other KWs - COVID-19 waves

(a) GHQ Likert (b) GHQ Caseness


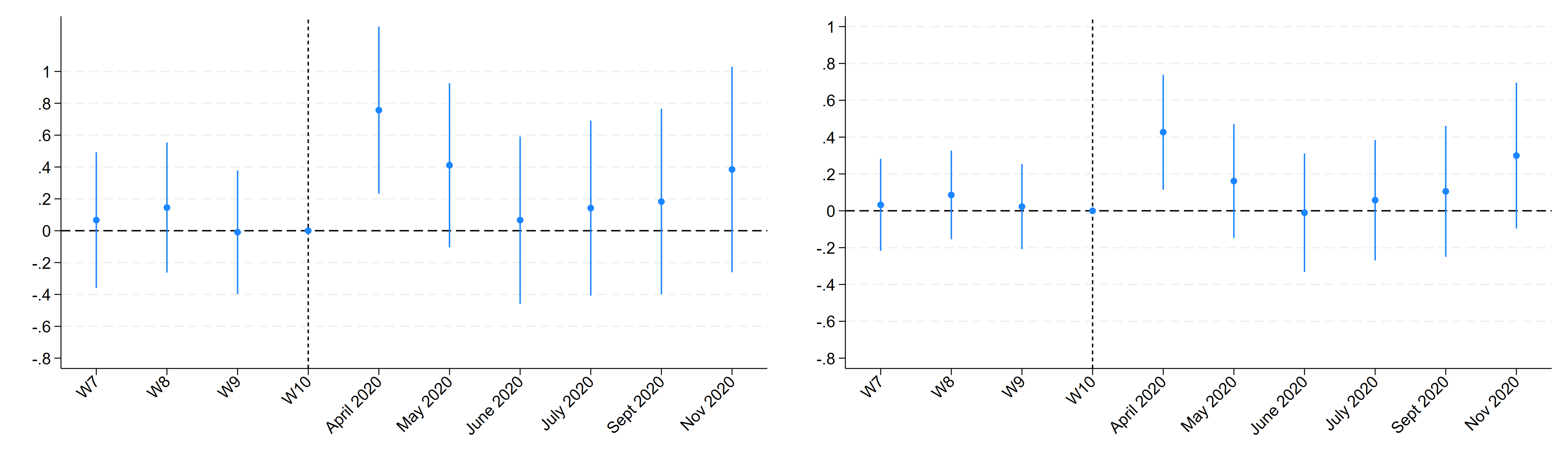


(c) Distress (d) Severe Distress


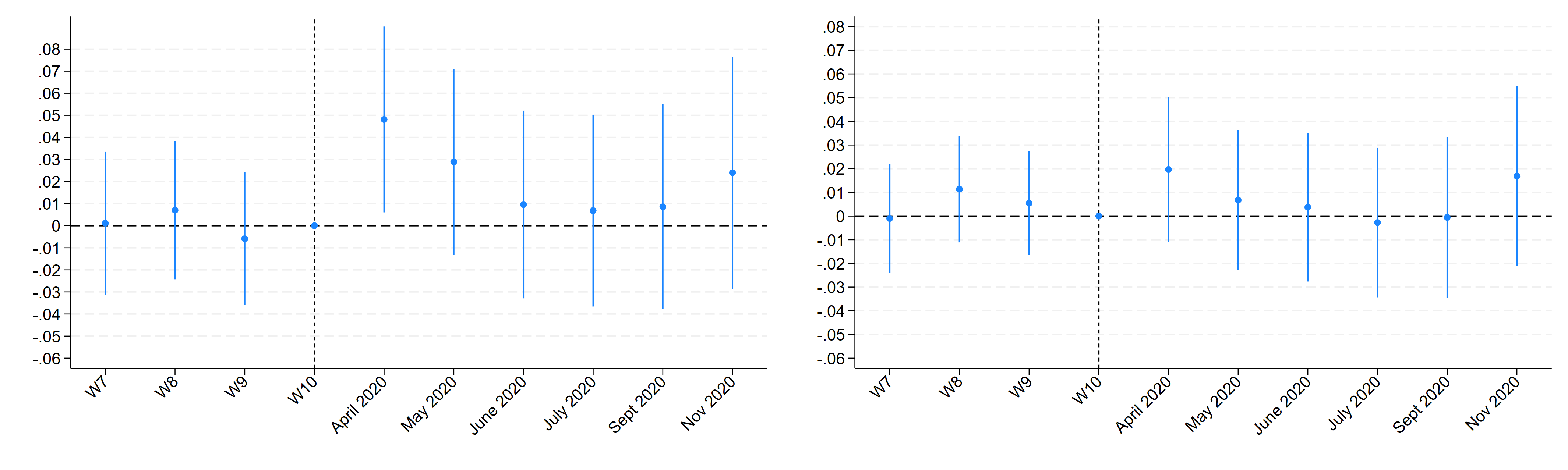


*Notes*: Regressions based on sample that includes waves 7 to 10 (pre-pandemic) and waves April to November 2020 (post-pandemic). Estimates derived from the DID model that interacts the worker group variable with indicators for waves. Wave 10 is the reference period. All model specifications incorporate IPWs to adjust for attrition. Controls included are age, education, marital status, number of children aged 0 to 15, household income, region dummies and interview year dummies. Estimates obtained using the unbalanced sample. Standard errors clustered at the household level. 95% confidence intervals shown.

### Figure A6: Heterogeneous effects HSC KWs vs Non-KWs - COVID-19 waves

(a) GHQ Likert (b) GHQ Caseness


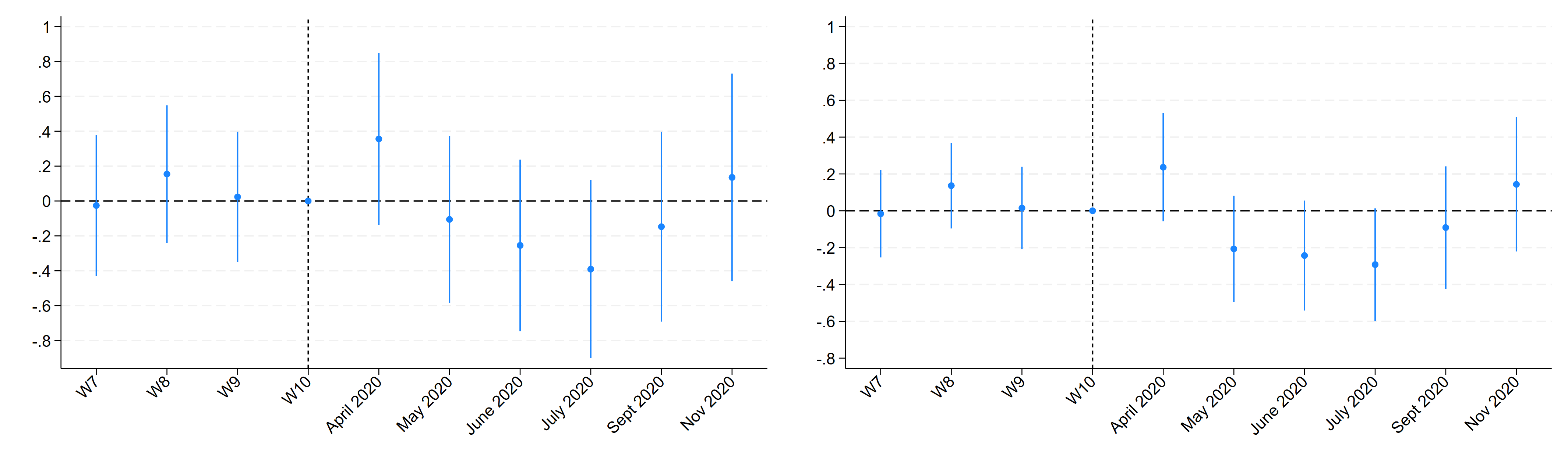


(c) Distress (d) Severe Distress


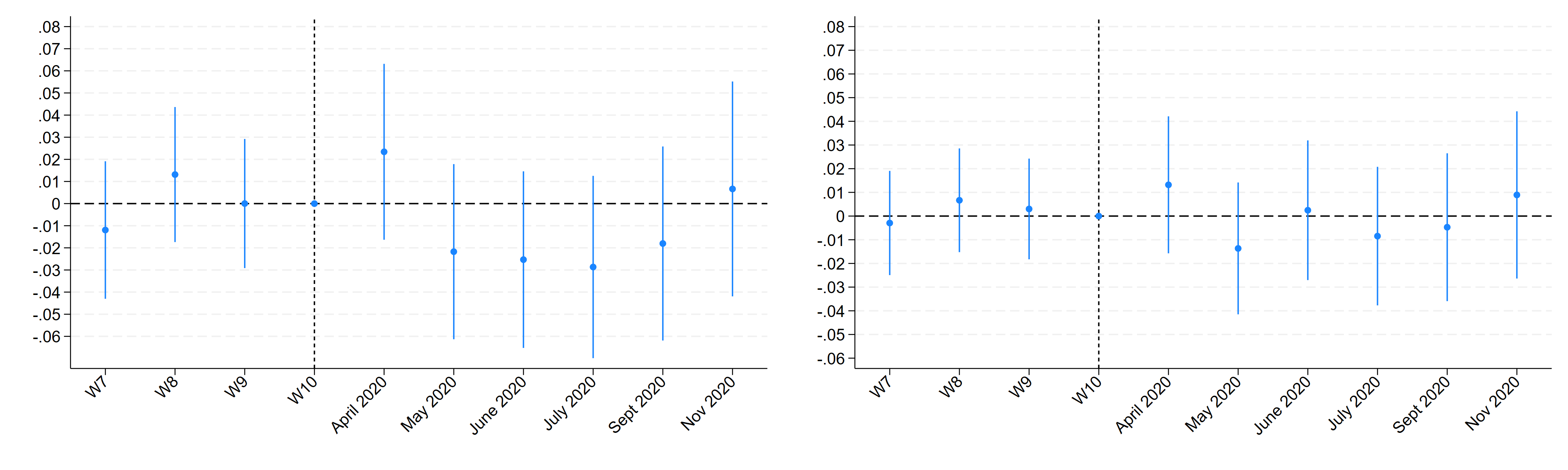


*Notes*: Regressions based on sample that includes waves 7 to 10 (pre-pandemic) and waves April to November 2020 (post-pandemic). Estimates derived from the DID model that interacts the worker group variable with indicators for waves. Wave 10 is the reference period. All model specifications incorporate IPWs to adjust for attrition. Controls included are age, education, marital status, number of children aged 0 to 15, household income, region dummies and interview year dummies. Estimates obtained using the unbalanced sample. Standard errors clustered at the household level. 95% confidence intervals shown.

### Figure A7: Heterogeneous effects HSC KWs vs Other KWs - Main waves

(a) GHQ Likert (b) GHQ Caseness


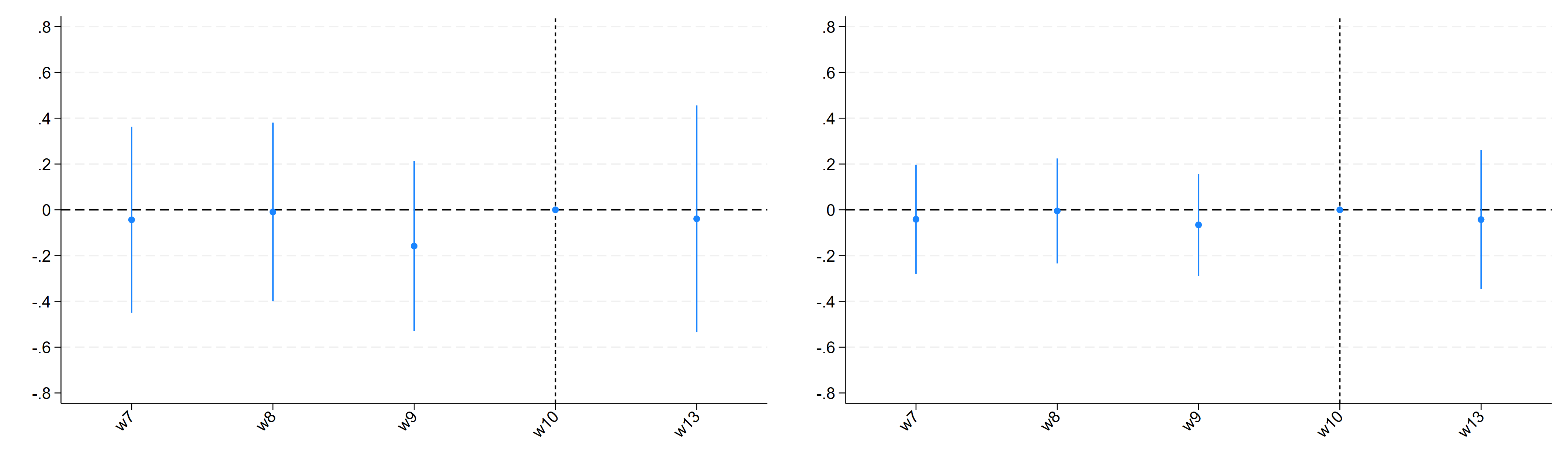


(c) Distress (d) Severe Distress


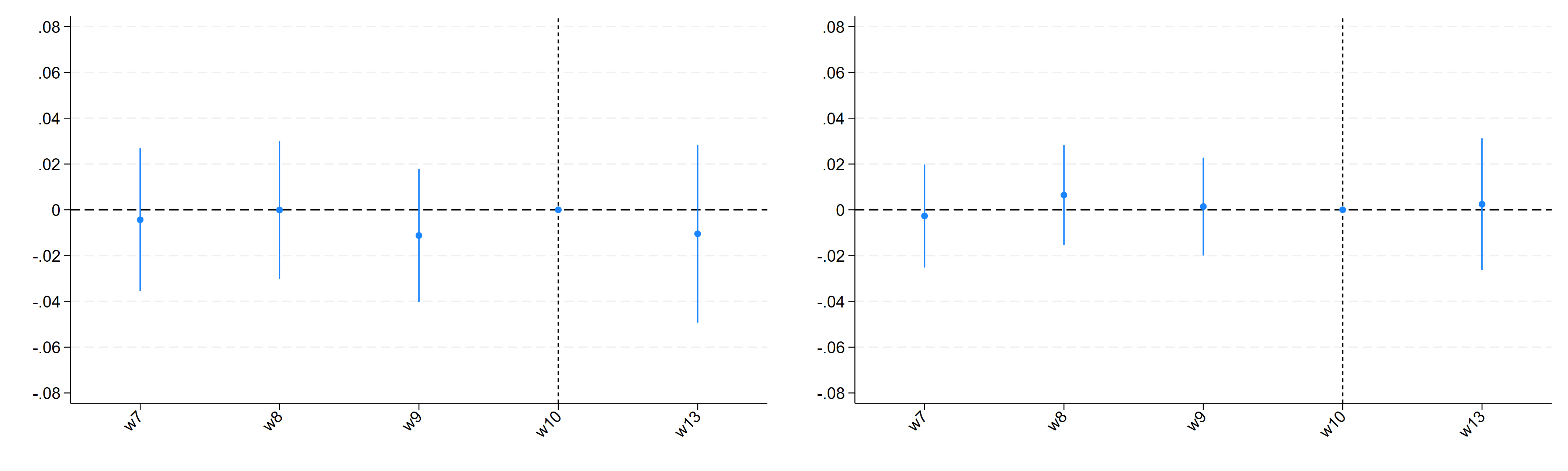


*Notes*: Regressions based on sample that includes waves 7 to 10 (pre-pandemic) and wave 13 (post-pandemic). Estimates derived from the DID model that interacts the worker group variable with indicators for waves. Wave 10 is the reference period. All model specifications incorporate IPWs to adjust for attrition. Controls included are age, education, marital status, number of children aged 0 to 15, household income, region dummies and interview year dummies. Estimates obtained using the unbalanced sample. Standard errors clustered at the household level. 95% confidence intervals shown.

### Figure A8: Heterogeneous effects HSC KWs vs Non-KWs - Main waves

(a) GHQ Likert (b) GHQ Caseness


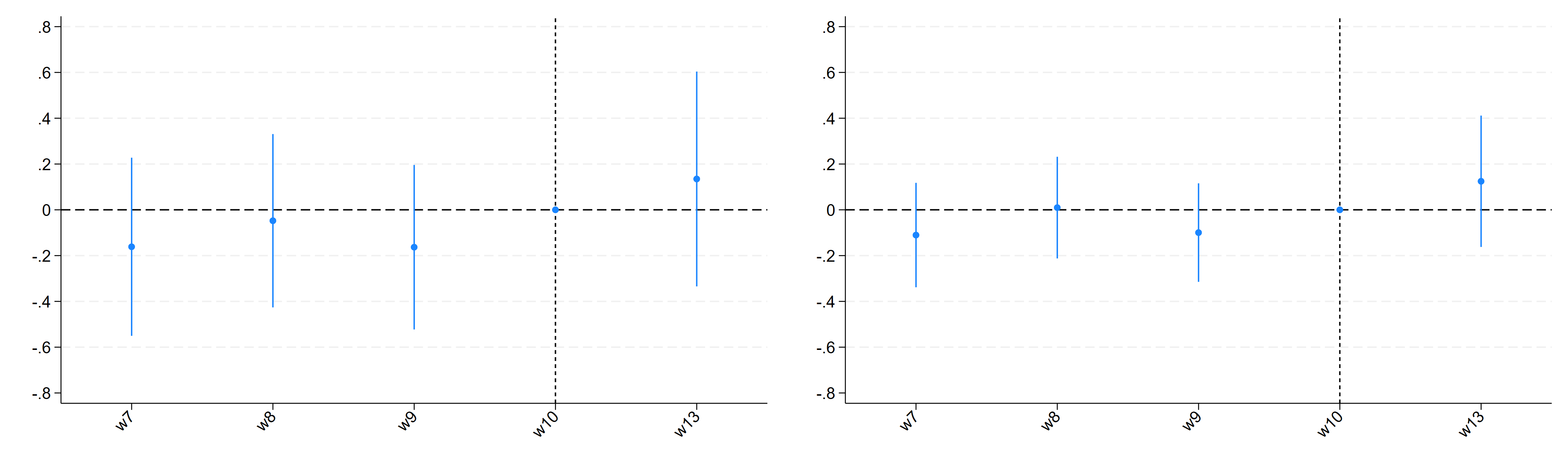


(c) Distress (d) Severe Distress


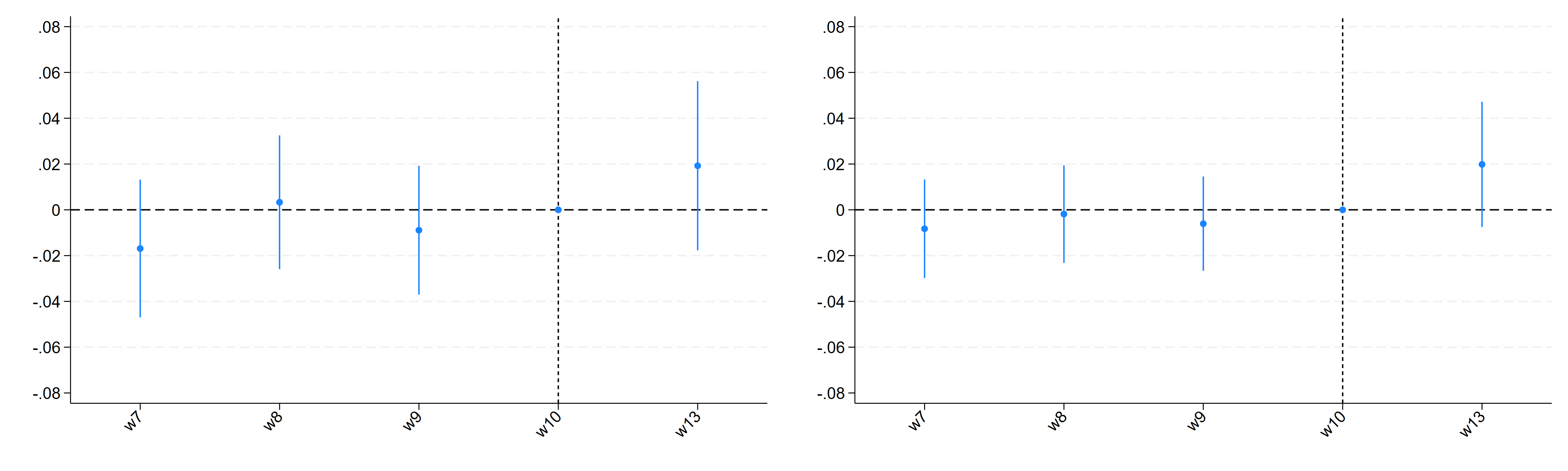


*Notes*: Regressions based on sample that includes waves 7 to 10 (pre-pandemic) and wave 13 (post-pandemic). Estimates derived from the DID model that interacts the worker group variable with indicators for waves. Wave 10 is the reference period. All model specifications incorporate IPWs to adjust for attrition. Controls included are age, education, marital status, number of children aged 0 to 15, household income, region dummies and interview year dummies. Estimates obtained using the unbalanced sample. Standard errors clustered at the household level. 95% confidence intervals shown.

### Figure A9: Heterogeneous effects HSC KWs vs Other KWs - Calendar Year data

(a) GHQ Likert (b) GHQ Caseness


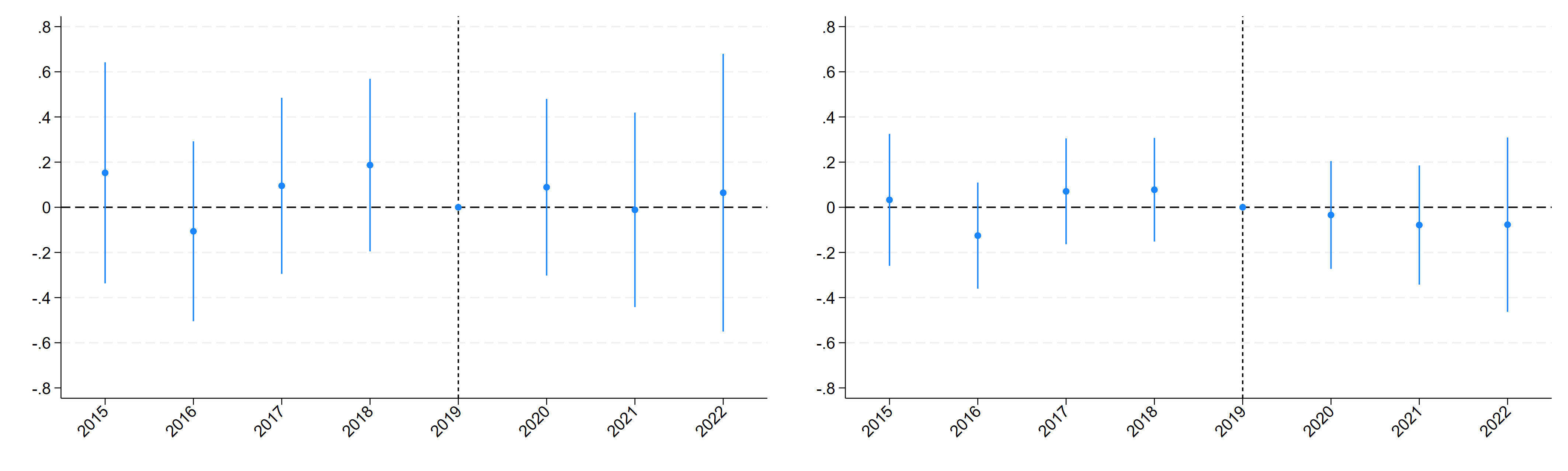


(c) Distress (d) Severe Distress


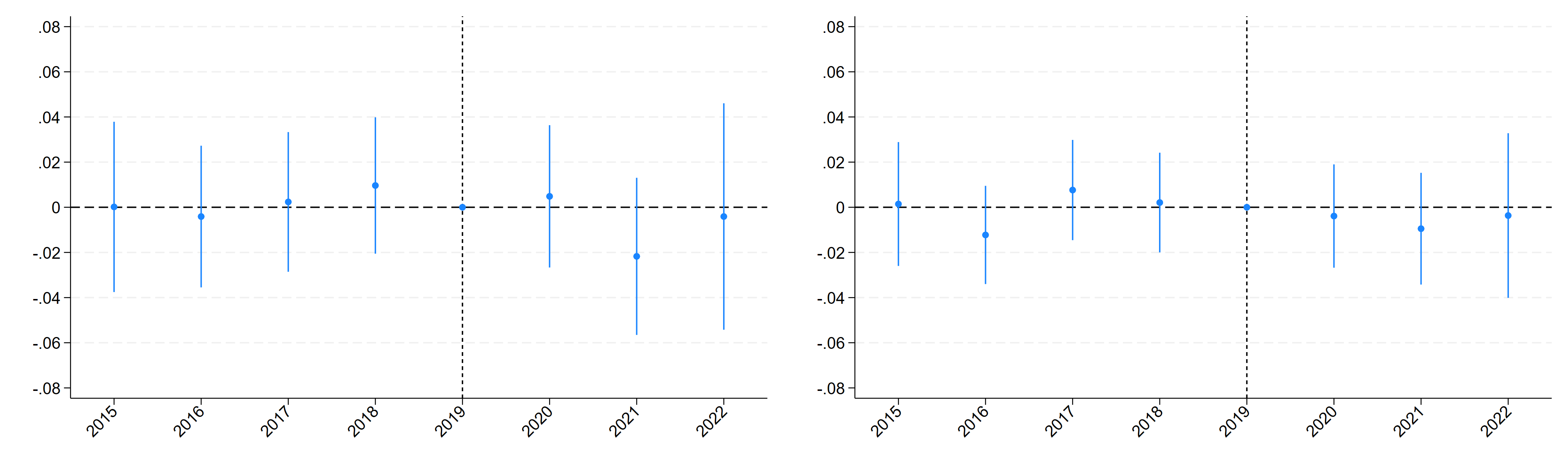


*Notes*: Regressions based on sample that use calendar year data (2015-2022). Estimates derived from the DID model that interacts the worker group variable with indicators for year. 2019 is the reference year. All model specifications incorporate IPWs to adjust for attrition. Controls included are age, education, marital status, number of children aged 0 to 15, household income, region dummies and interview year dummies. Estimates obtained using the unbalanced sample. Standard errors clustered at the household level. 95% confidence intervals shown.

### Figure A10: Heterogeneous effects HSC KWs vs Non-KWs - Calendar Year data

(a) GHQ Likert (b) GHQ Caseness


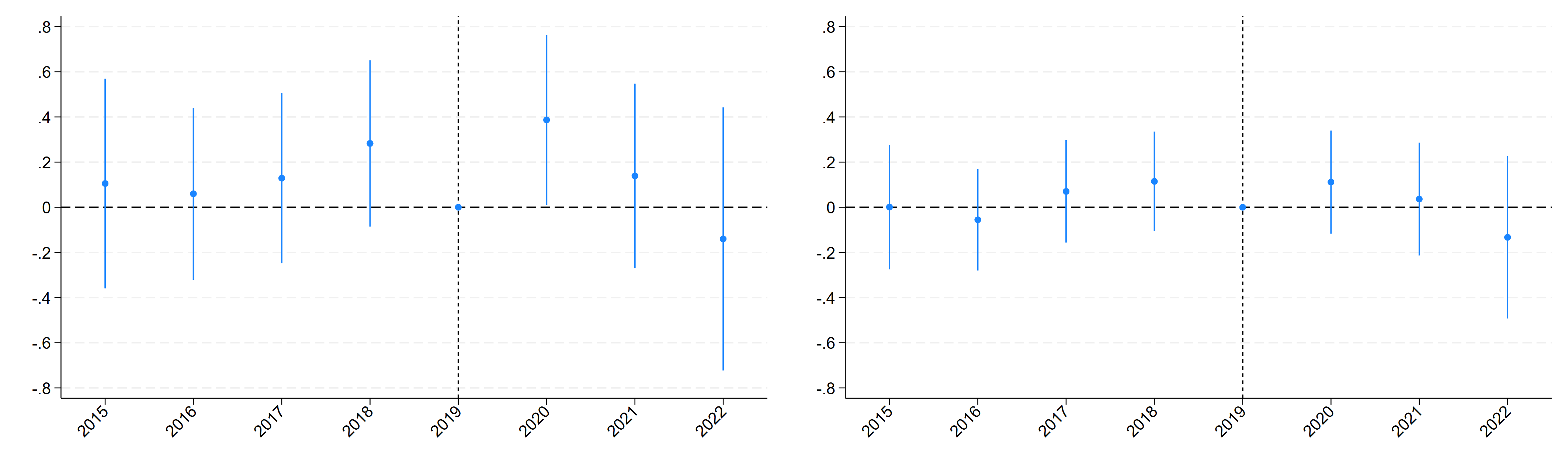


(c) Distress (d) Severe Distress


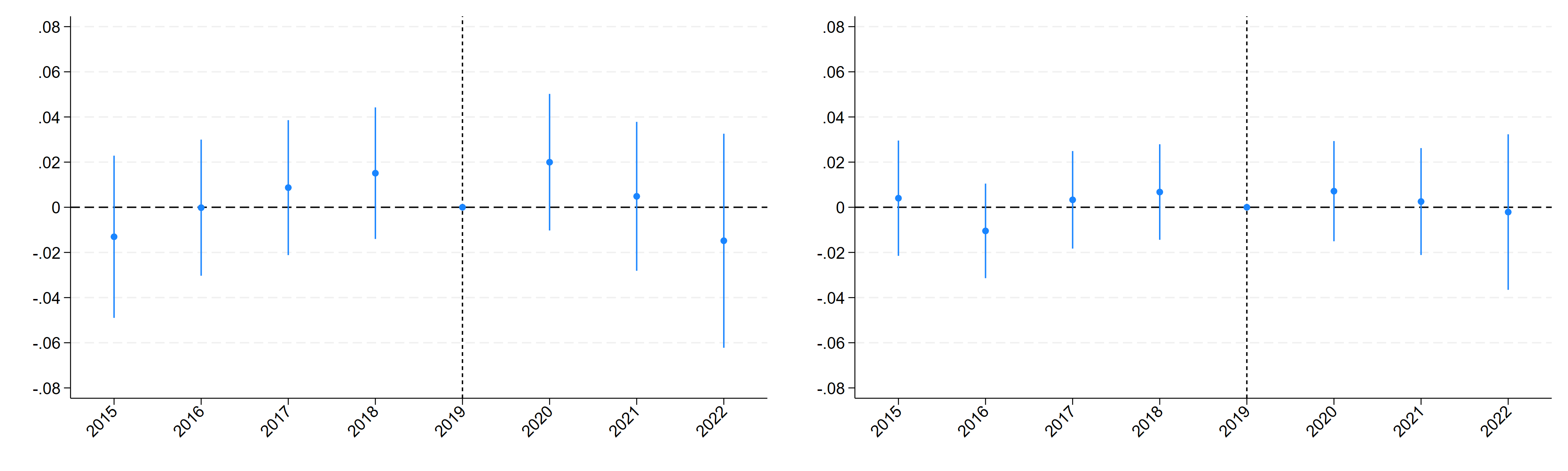


*Notes*: Regressions based on sample that use calendar year data (2015-2022). Estimates derived from the DID model that interacts the worker group variable with indicators for year. 2019 is the reference year. All model specifications incorporate IPWs to adjust for attrition. Controls included are age, education, marital status, number of children aged 0 to 15, household income, region dummies and interview year dummies. Estimates obtained using the unbalanced sample. Standard errors clustered at the household level. 95% confidence intervals shown.

### Figure A11: Mechanisms - HSC KWs vs Other KWs

(a) Current Finances (b) Future Finances


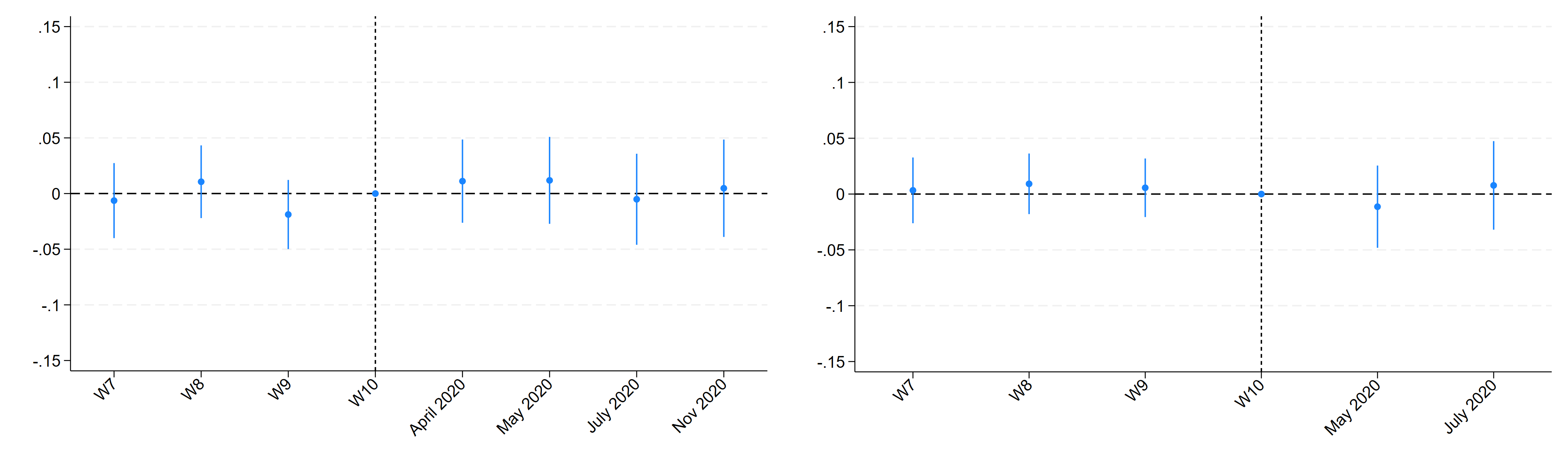


(c) Loneliness


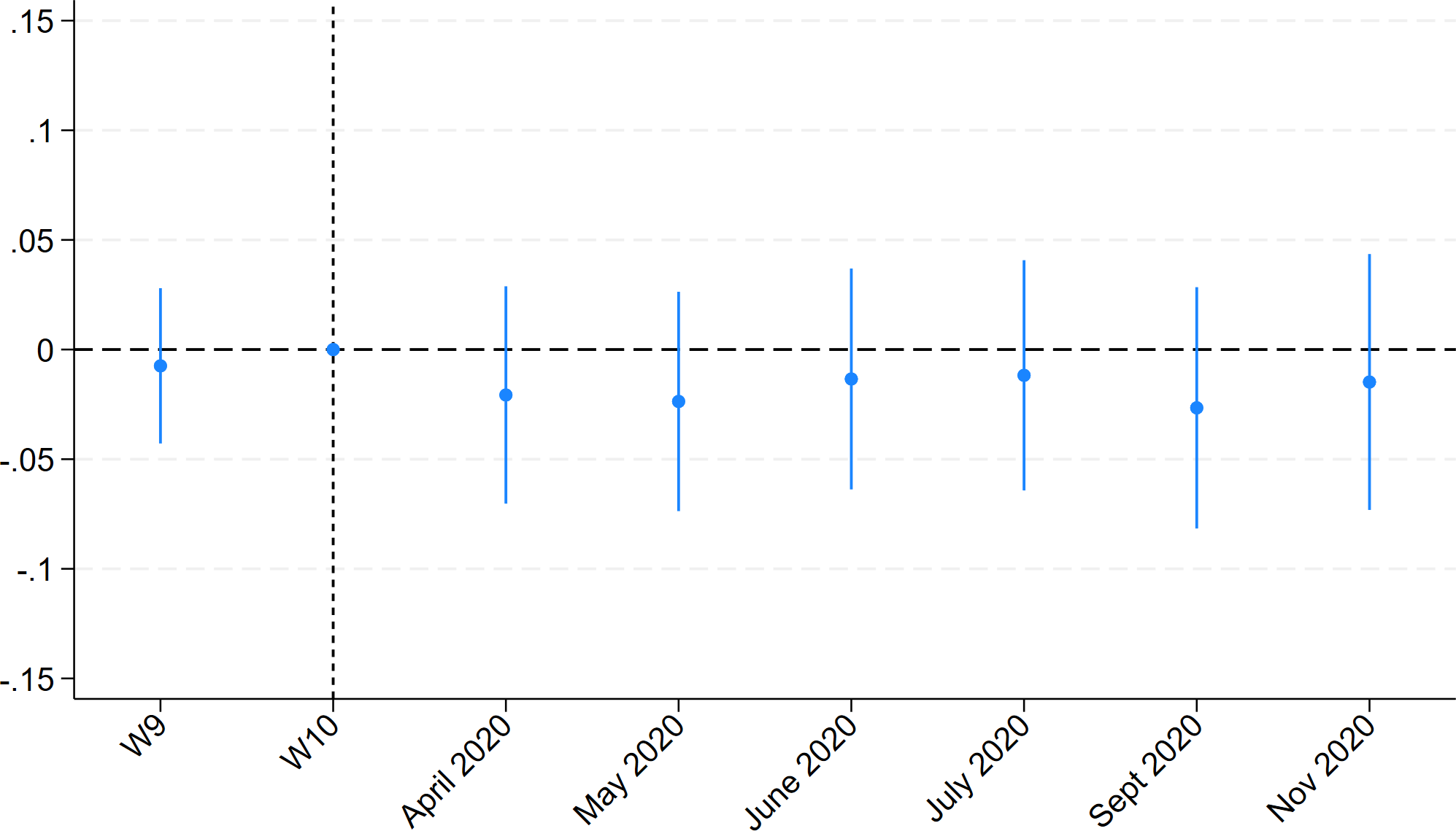


*Notes*: Regressions based on sample that includes waves 7 to 10 (pre-pandemic) and waves April to November 2020 (post-pandemic). Estimates derived from the DID model that interacts the worker group variable with indicators for waves. Wave 10 is the reference period. Outcome variable on current financial stability is available in waves April, May, July and November 2020. Outcome variable on future financial stability is available for May and July 2020. Outcome variable on loneliness is available for all wave between April and November July 2020. All model specifications incorporate IPWs to adjust for attrition. Controls included are age, education, marital status, number of children aged 0 to 15, household income, region dummies and interview year dummies. Estimates obtained using the unbalanced sample. Standard errors clustered at the household level. 95% confidence intervals shown.

### Figure A12: Mechanisms - HSC KWs vs Non-KWs

(a) Current Finances (b) Future Finances


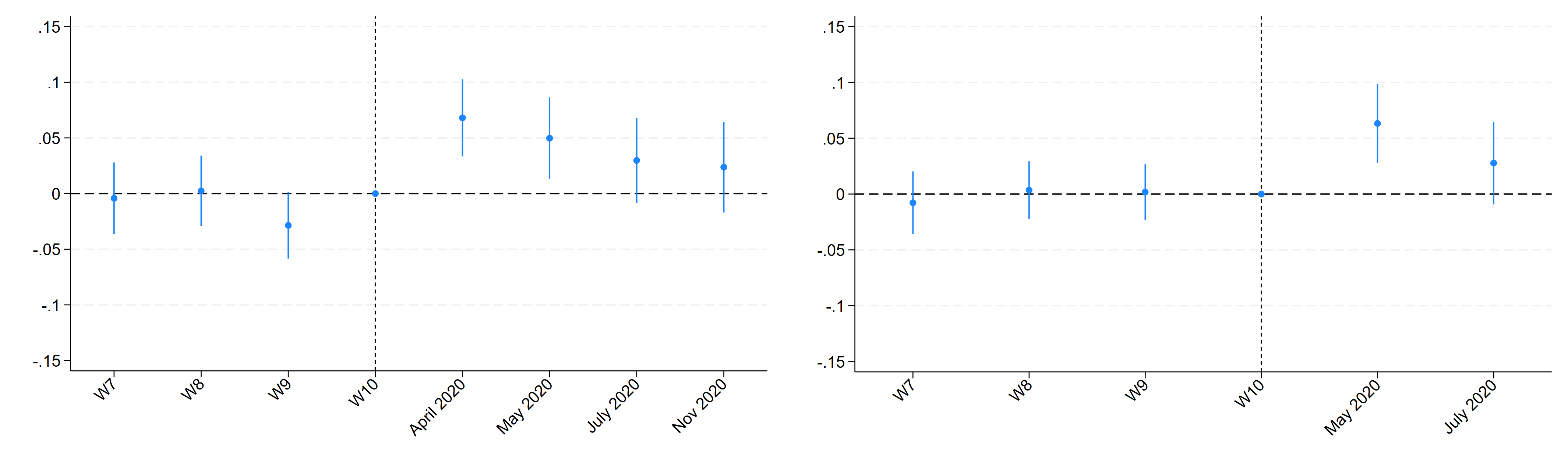


(c) Loneliness


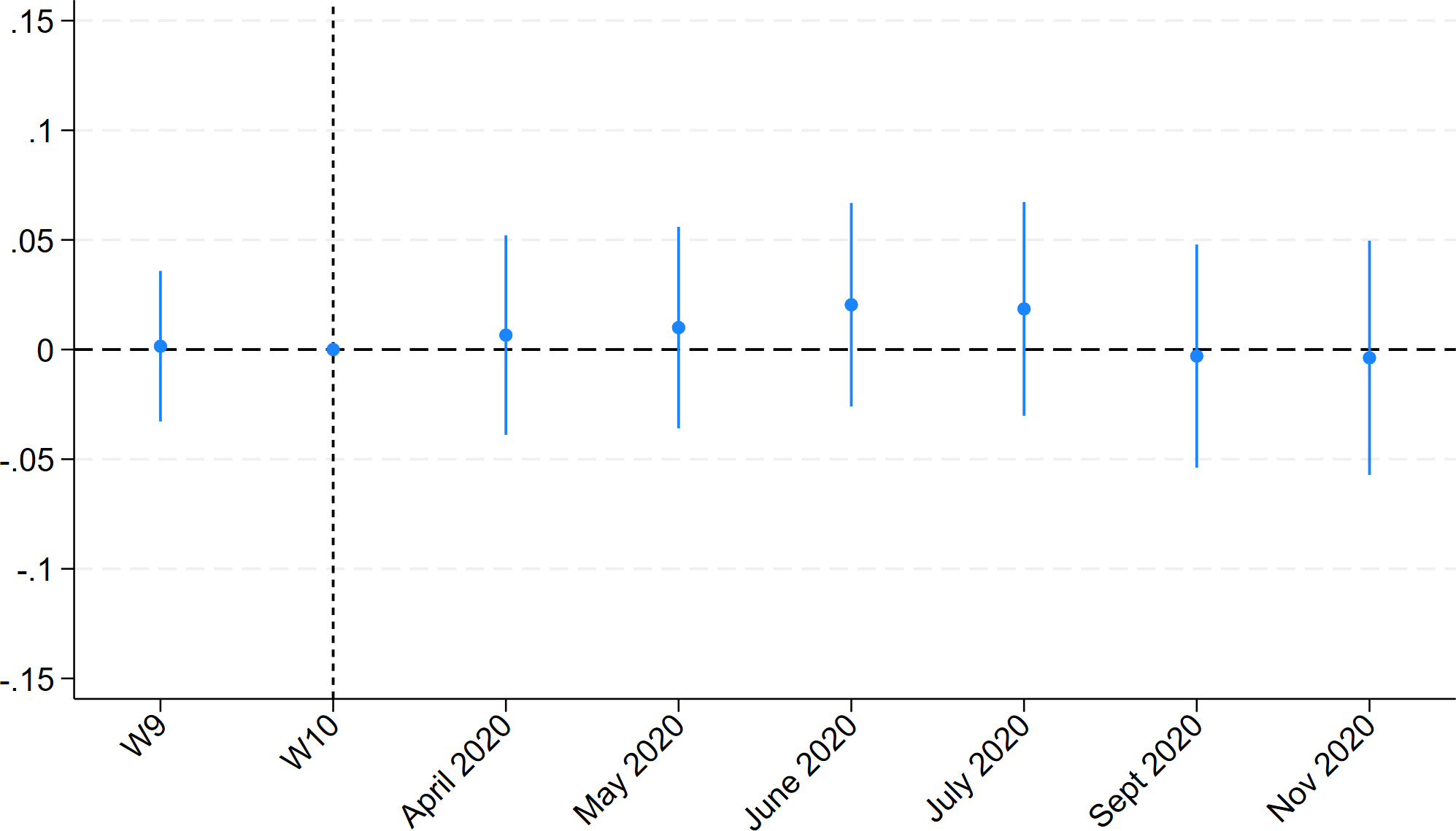


*Notes*: Regressions based on sample that includes waves 7 to 10 (pre-pandemic) and waves April to November 2020 (post-pandemic). Estimates derived from the DID model that interacts the worker group variable with indicators for waves. Wave 10 is the reference period. Outcome variable on current financial stability is available in waves April, May, July and November 2020. Outcome variable on future financial stability is available for May and July 2020. Outcome variable on loneliness is available for all wave between April and November July 2020. All model specifications incorporate IPWs to adjust for attrition. Controls included are age, education, marital status, number of children aged 0 to 15, household income, region dummies and interview year dummies. Estimates obtained using the unbalanced sample. Standard errors clustered at the household level. 95% confidence intervals shown.
